# Overview of emerging nonvolatile memory technologies

**DOI:** 10.1186/1556-276X-9-526

**Published:** 2014-09-25

**Authors:** Jagan Singh Meena, Simon Min Sze, Umesh Chand, Tseung-Yuen Tseng

**Affiliations:** 1 Department of Electronics Engineering and Institute of Electronics, National Chiao Tung University, Hsinchu 30010, Taiwan

**Keywords:** Emerging nonvolatile memory technologies, Magnetic storage, Market memory technologies, Memristors, Phase change memories, Random-access storage, Flash memory technologies, Three-dimensional memory, Transparent memory, Unified memory

## Abstract

Nonvolatile memory technologies in Si-based electronics date back to the 1990s. Ferroelectric field-effect transistor (FeFET) was one of the most promising devices replacing the conventional Flash memory facing physical scaling limitations at those times. A variant of charge storage memory referred to as Flash memory is widely used in consumer electronic products such as cell phones and music players while NAND Flash-based solid-state disks (SSDs) are increasingly displacing hard disk drives as the primary storage device in laptops, desktops, and even data centers. The integration limit of Flash memories is approaching, and many new types of memory to replace conventional Flash memories have been proposed. Emerging memory technologies promise new memories to store more data at less cost than the expensive-to-build silicon chips used by popular consumer gadgets including digital cameras, cell phones and portable music players. They are being investigated and lead to the future as potential alternatives to existing memories in future computing systems. Emerging nonvolatile memory technologies such as magnetic random-access memory (MRAM), spin-transfer torque random-access memory (STT-RAM), ferroelectric random-access memory (FeRAM), phase-change memory (PCM), and resistive random-access memory (RRAM) combine the speed of static random-access memory (SRAM), the density of dynamic random-access memory (DRAM), and the nonvolatility of Flash memory and so become very attractive as another possibility for future memory hierarchies. Many other new classes of emerging memory technologies such as transparent and plastic, three-dimensional (3-D), and quantum dot memory technologies have also gained tremendous popularity in recent years. Subsequently, not an exaggeration to say that computer memory could soon earn the ultimate commercial validation for commercial scale-up and production the cheap plastic knockoff. Therefore, this review is devoted to the rapidly developing new class of memory technologies and scaling of scientific procedures based on an investigation of recent progress in advanced Flash memory devices.

## Review

### Background

#### ***General overview***

The idea of using a floating gate (FG) device to obtain a nonvolatile memory device was suggested for the first time in 1967 by Kahng D and Sze SM at Bell Labs [1]. This was also the first time that the possibility of nonvolatile MOS memory device was recognized. From that day, semiconductor memory has made tremendous contributions to the revolutionary growth of digital electronics since a 64-bit bipolar RAM chip to be used in the cache memory of an IBM computer was reported in 1969 [2]. Semiconductor memory has always been an indispensable component and backbone of modern electronic systems. All familiar computing platforms ranging from handheld devices to large supercomputers use storage systems for storing data temporarily or permanently [3]. Beginning with punch card which stores a few bytes of data, storage systems have reached to multiterabytes of capacities in comparatively less space and power consumption. Regarding application aspects, the speed of storage systems needs to be as fast as possible [4]. Since Flash memory has become a common component of solid-state disks (SSDs), the falling prices and increased densities have made it more cost-effective for many other applications [5]. Memory devices and most SSDs that use Flash memory are likely to serve very different markets and purposes. Each has a number of different attributes which are optimized and adjusted to best meet the needs of particular users. Because of natural inherent limitations, the long-established memory devices have been shorted out according to their inventions to match with portable electronic data storage systems. Today, the most prominent one is the limited capacity for continued scaling of the electronic device structure. Research is moving along the following paths for embedded Flash devices: (i) scaling down the cell size of device memory, (ii) lowering voltage operation, and (iii) increasing the density of state per memory cell by using a multilevel cell. To sustain the continuous scaling, conventional Flash devices may have to undergo revolutionary changes. Basically, it is expected that an entire DVD collection be in the palm of a hand. Novel device concepts with new physical operationing principles are needed. It is worthwhile to take a look at semiconductor memories against the background of digital systems. The way semiconductor devices are used in a systems environment determines what is required of them in terms of density, speed/power, and functions. It is also worthwhile to look into the economic significance of semiconductor memories and the relative importance of their various types. For the past three and a half decades in existence, the family of semiconductor memories has expanded greatly and achieved higher densities, higher speeds, lower power, more functionality, and lower costs [3,6,7]. At the same time, some of the limitations within each type of memory are also becoming more realized. As such, there are several emerging technologies aiming to go beyond those limitations and potentially replace all or most of the existing semiconductor memory technologies to become a universal semiconductor memory (USM). In addition, the rewards for achieving such a device would be to gain control of an enormous market, which has expanded from computer applications to all of consumer electronic products. Looking forward to the future, there are wide ranges of emerging memory applications for automation and information technology to health care. The specification of nonvolatile memory (NVM) is based on the floating gate configuration, which is the feature of an erased gate put into many cells to facilitate block erasure. Among them, designed Flash memories such as NOR and NAND Flash have been developed and then proposed as commercial products into bulk market. They have been considered as the most important products. NOR has high operation speed for both code and data storage applications; on the other hand, NAND has high density for large data storage applications [8]. Since the inception of Flash memory, there has been an exponential growth in its market driven primarily by cell phones and other types of consumer electronic equipment. While, today, integration of a silicon chip is not economical, toys, cards, labels, badges, value paper, and medical disposables could be imagined to be equipped with flexible electronics and memory. With growing demands for high-density digital information storage, memory density with arriving technology has been increased dramatically from the past couple of years. The main drive to develop organic nonvolatile memory is currently for applications of thin-film, flexible, or even printed electronics. One needs a technology to tag everything to electronic functionality which can be foreseen in a very large quantity and at a very low cost on substrates such as plastic and paper. Accessible popularization of roll-to-roll memory commercialization is a way to make an encounter interesting and challenging to have charge storage devices of choice for applications with enormous flexibility and strength. Recently, polymer (plastic memory) and organic memory devices have significant consideration because of their simple processes, fast operating speed, and excellent switching ability [9,10]. One significant advantage polymer memory has over conventional memory designs is that it can be stacked vertically, yielding a three-dimensional (3-D) use of space [11]. This means that in terabyte solid-state devices with extremely low transistor counts such as drives about the size of a matchbook, the data persists even after power is removed. The NAND Flash market is continually growing by the successive introduction of innovative devices and applications. To meet the market trend, 3-D NVMs are expected to replace the planar ones, especially for 10-nm nodes and beyond. Moreover, simple-structure organic bistable memory exhibiting superior memory features has been realized by employing various nanoparticles (NPs) blended into a single-layered organic material sandwiched between two metal electrodes [12,13]. The NPs act as traps that can be charged and discharged by suitable voltage pulses. NP blends show promising data retention times, switching speed, and cycling endurance, but the on-state current is too low to permit scaling to nanometer dimensions [10,14]. A lot of these great ideas tend to die before reaching this point of development, but that is not to say that we will be seeing plastic memory on store shelves next year. There are still many hurdles to get over; software alone is a big task, as is the manufacturing process, but it does bring this technology one step closer to reality [15]. It is not an exaggeration to say that the equivalent of 400,000 CDs, 60,000 DVDs, or 126 years of MPG music may be stored on a polymer memory chip the size of a credit card.

#### ***The vision of this review***

In this review, we focus on electrically programmable nonvolatile memory changes from silicon nanocrystal memory scaling to organic and metallic NP memory devices. Further, the scaling trend move towards the emerging NVM to flexible and transparent redox-based resistive switching memory technologies. This review is intended to give an overview to the reader of storage systems and components from conventional memory devices that have been proposed in the past years of recent progress in current NVM devices based on nanostructured materials to redox-based resistive random-access memory (RRAM) to 3-D and transparent memory devices. We describe the basics of Flash memory and then highlight the present problems with the issue of scaling tunnel dielectric in these devices. We briefly describe a historical change, how the conventional FG nonvolatile memory suffers from a charge loss problem as the feature size of the device continues to shrink. A discrete polysilicon-oxide-nitride-oxide-silicon (SONOS) memory is then proposed as a replacement of the conventional FG memory. The NC memory is expected to efficiently preserve the trapped charge due to the discrete charge storage node while also demonstrating excellent features such as fast program/erase speeds, low programming potentials, and high endurance. We also discuss current ongoing research in this field and the solutions proposed to solve the scaling problems by discussing a specific solution in detail which would be the centerpiece in recent memory work progress. Moreover, this review makes distinct emerging memory concepts with more recent molecular and quantum dot programmable nonvolatile memory concepts, specifically using charge trapping in conjugated polymers and metal NPs. We classify several possible devices, according to their operating principle, and critically review the role of π-conjugated materials in the data storage device operation. We describe specifications for applications of emerging NVM devices as well as already existing NAND memory and review the state of the art with respect to these target specifications in the future. Conclusions are drawn regarding further work on materials and upcoming memory devices and architectures.

### Classification of solid-state memory technologies

**Figure 1:**
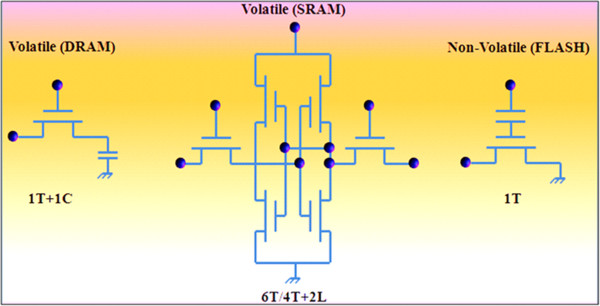
The circuitry structures of DRAM, SRAM, and Flash memories.

Data storage devices can be classified based on many functional criteria. Of them, silicon-based semiconductor memories are categorized into two: volatile and nonvolatile [3,16]. In volatile memories, the information eventually fades while power supply is turned off unless the devices used to store data will be periodically refreshed. On the other hand, nonvolatile memories retain the stored information even when the power supply is turned off. Volatile memories, such as static random-access memory (SRAM) and dynamic random-access memory (DRAM), need voltage supply to hold their information while nonvolatile memories, namely Flash memories, hold their information without one. DRAM (dynamic stands for the periodical refresh) is needed for data integrity in contrast to SRAM. The basic circuit structures of DRAM, SRAM, and Flash memories are shown in Figure 1. DRAM, SRAM, and Flash are today's dominant solid-state memory technologies, which have been around for a long time, with Flash the youngest, at 25 years. DRAM is built using only one transistor and one capacitor component, and SRAM is usually built in CMOS technology with six transistors. Two cross-coupled inverters are used to store the information like in a flip-flop. For the access control, two further transistors are needed. If the write line is enabled, then data can be read and set with the bit lines. The Flash memory circuit works with the FG component. The FG is between the gate and the source-drain area and isolated by an oxide layer. If the FG is uncharged, then the gate can control the source-drain current. The FG gets filled (tunnel effect) with electrons when a high voltage at the gate is supplied, and the negative potential on the FG works against the gate and no current is possible. The FG can be erased with a high voltage in reverse direction of the gate. DRAM has an advantage over SRAM and Flash of only needing one MOSFET with a capacitor. It also has the advantage of cheap production as well as lower power consumption as compared to SRAM but slower than SRAM. On the other hand, SRAM is usually built in CMOS technology with six transistors and two cross-coupled inverters, and for the access control, two further transistors are needed. SRAM has the advantage of being quick, easy to control, integrated in the chip, as well as fast because no bus is needed like in DRAM. But SRAM has the disadvantages of needing many transistors and hence expensive, higher power consumption than DRAM. In comparison to DRAM and SRAM, Flash memory has FG between the gate and the source-drain area and isolated with an oxide layer. Flash memory does not require power to store information but is slower than SRAM and DRAM.

**Figure 2:**
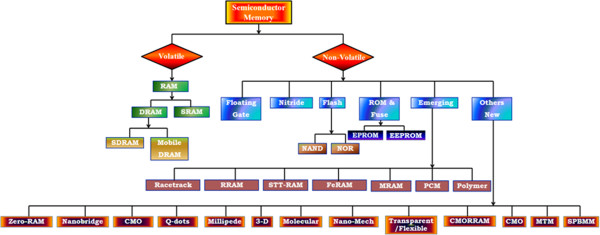
Flow chart for the semiconductor memory classification according to their functional criteria.

Both types of memories can be further classified based on the memory technology that they use and based on data volatility as shown in the classification flow chart depicted in Figure 2. Volatile memories consist mostly of DRAM [17], which can be further classified into SDRAM and mobile RAM which only retain information when current is constantly supplied to the device [18]. Another small but very important memory device is SRAM. The market for DRAM devices far exceeds the market for SRAM devices, although a small amount of SRAM devices is used in almost all logic and memory chips. However, DRAM uses only one transistor and one capacitor per bit, allowing it to reach much higher densities and, with more bits on a memory chip, be much cheaper per bit. SRAM is not worthwhile for desktop system memory, where DRAM dominates, but is used for its cache memories. SRAM is commonplace in small embedded systems, which might only need tens of kilobytes or less. Forthcoming volatile memory technologies that hope to replace or compete with SRAM and DRAM include Z-RAM, TTRAM, A-RAM, and ETA RAM. In the industry, new universal and stable memory technologies will appear as real contenders to displace either or both NAND Flash and DRAM. Flash memory is presently the most suitable choice for nonvolatile applications for the following reasons: Semiconductor nonvolatile memories consist mostly of the so-called ‘Flash’ devices and retain their information even when the power is turned off. Other nonvolatile semiconductor memories include mask read-only memory (MROM), antifuse-based one-time programmable (OTP) memory, and electrically erasable read-only memory (EEPROM). Flash is further divided into two categories: NOR, characterized by a direct write and a large cell size, and NAND, characterized by a page write and small cell size. Nonvolatile memory is a computer memory that can retain the stored information even when not powered [3,19,20]. Nonvolatile semiconductor memories are generally classified according to their functional properties with respect to the programming and erasing operations, as shown in the flow chart described in Figure 2. These are floating gate, nitride, ROM and fuse, Flash, emerging, and other new next-generation memory technologies. Today, these nonvolatile memories are highly reliable and can be programmed using a simple microcomputer and virtually in every modern electronic equipment, which are expected to replace existing memories.

Among them, emerging nonvolatile memories are now very captivating. The next-generation memory market will cover up these emerging memory technologies [21]. There are mainly five types of nonvolatile memory technology: Flash memory, ferroelectric random-access memory (FeRAM), magnetic random-access memory (MRAM), phase-change memory (PCM), and RRAM. Nonvolatile memory, specifically ‘Flash’ memory, which is characterized by a large-block (or ‘sector’) erasing mechanism, has been the fastest growing segment of the semiconductor business for the last 10 years. Some of these newer emerging technologies include MRAM, FeRAM, PCM, spin-transfer torque random-access memory (STT-RAM), RRAM and memristor. MRAM is a nonvolatile memory [10,22]. Unlike DRAM, the data is not stored in an electric charge flow, but by magnetic storage elements. The storage elements are formed by two ferromagnetic plates, each of which can hold a magnetic field, separated by a thin insulating layer. One of the two plates is a permanent magnet set to a particular polarity; the other's field can be changed to match that of an external field to store memory. STT-RAM is an MRAM (nonvolatile) but with better scalability over traditional MRAM. The STT is an effect in which the orientation of a magnetic layer in a magnetic tunnel junction or spin valve can be modified using a spin-polarized current. Spin-transfer torque technology has the potential to make MRAM devices combining low current requirements and reduced cost possible; however, the amount of current needed to reorient the magnetization is at present too high for most commercial applications. PCM is a nonvolatile random-access memory, which is also called ovonic unified memory (OUM), based on reversible phase conversion between the amorphous and the crystalline state of a chalcogenide glass, which is accomplished by heating and cooling of the glass. It utilizes the unique behavior of chalcogenide (a material that has been used to manufacture CDs), whereby the heat produced by the passage of an electric current switches this material between two states. The different states have different electrical resistance which can be used to store data. The ideal memory device or the so-called unified memory would satisfy simultaneously three requirements: high speed, high density, and nonvolatility (retention). At the present time, such memory has not been developed. The floating gate nonvolatile semiconductor memory (NVSM) has high density and retention, but its program/erase speed is low. DRAM has high speed (approximately 10 ns) and high density, but it is volatile. On the other hand, SRAM has very high speed (approximately 5 ns) but limited from very low density and volatility. It is expected that PCM will have better scalability than other emerging technologies. RRAM is a nonvolatile memory that is similar to PCM. The technology concept is that a dielectric, which is normally insulating, can be made to conduct through a filament or conduction path formed after application of a sufficiently high voltage. Arguably, this is a memristor technology and should be considered as potentially a strong candidate to challenge NAND Flash. Currently, FRAM, MRAM, and PCM are in commercial production but still, relative to DRAM and NAND Flash, remain limited to niche applications. There is a view that MRAM, STT-RAM, and RRAM are the most promising emerging technologies, but they are still many years away from competing for industry adoption [23]. Any new technology must be able to deliver most, if not all, of the following attributes in order to drive industry adoption on a mass scale: scalability of the technology, speed of the device, and power consumption to be better than existing memories. The NVSM is in inspiring search of novel nonvolatile memories, which will successfully lead to the realization and commercialization of the unified memory.

In progress, another new class of nonvolatile memory technologies will offer a large increase in flexibility compared to disks, particularly in their ability to perform fast, random accesses. Unlike Flash memory, these new technologies will support in-place updates, avoiding the extra overhead of a translation layer. Further, these new nonvolatile memory devices based on deoxyribonucleic acid (DNA) biopolymer and organic and polymer materials are one of the key devices for the next-generation memory technology with low cost. Nonvolatile memory based on metallic NPs embedded in a polymer host has been suggested as one of these new cross-point memory structures. In this system, trap levels situated within the bandgap of the polymer are introduced by the NPs [24,25]. Memory devices play a massive role in all emerging technologies; as such, efforts to fabricate new organic memories to be utilized in flexible electronics are essential. Flexibility is particularly important for future electronic applications such as affordable and wearable electronics. Much research has been done to apply the flexible electronics technology to practical device areas such as solar cells, thin-film transistors, photodiodes, light-emitting diodes, and displays [26-28]. Research on flexible memory was also initiated for these future electronic applications. In particular, organic-based flexible memories have merits such as a simple, low-temperature, and low-cost manufacturing process. Several fabrication results of organic resistive memory devices on flexible substrates have been reported [29,30]. In addition, with growing demand for high-density digital information storage, NAND Flash memory density has been increased dramatically for the past couple of decades. On the other hand, device dimension scaling to increase memory density is expected to be more and more difficult in a bit-cost scalable manner due to various physical and electrical limitations. As a solution to the problems, NAND Flash memories having stacked layers are under developing extensions [31,32]. In 3-D memories, cost can be reduced by building multiple stacked cells in vertical direction without device size scaling. As a breakthrough for the scaling limitations, various 3-D stacked memory architectures are under development and expecting the huge market of 3-D memories in the near future. With lots of expectation, future-generation memories have potential to replace most of the existing memory technologies. The new and emerging memory technologies are also named to be a universal memory; this may give rise to a huge market for computer applications to all the consumer electronic products.

### Market memory technologies by applications

The semiconductor industry has experienced many changes since Flash memory first appeared in the early 1980s. The growth of consumer electronics market urges the demand of Flash memory and helps to make it a prominent segment within the semiconductor industry. The Flash memories were commercially introduced in the early 1990s, and since that time, they have been able to follow Moore's law and the scaling rules imposed by the market. There are expected massive changes in the memory market over the next couple of years, with more density and reliable technologies challenging the dominant NAND Flash memory now used in SSDs and embedded in mobile products. Server, storage, and application vendors are now working on new specifications to optimize the way their products interact with NVM - moves that could lead to the replacement of DRAM and hard drives alike for many applications, according to a storage networking industry association (SNIA) technical working group [33,34]. The Flash memory marketplace is one of the most vibrant and exciting in the semiconductor industry, not to mention one of the most competitive. The continuous invention of new memory technologies and their applications in the memory market also increase performance demands. These new classes of memories with the latest technology increase the vertical demand in the future memory market. In the next coming years, cumulative price reductions should become disruptive to DVDs and hard disk drives (HDDs), stimulate huge demand, and create new Flash markets.

**Figure 3:**
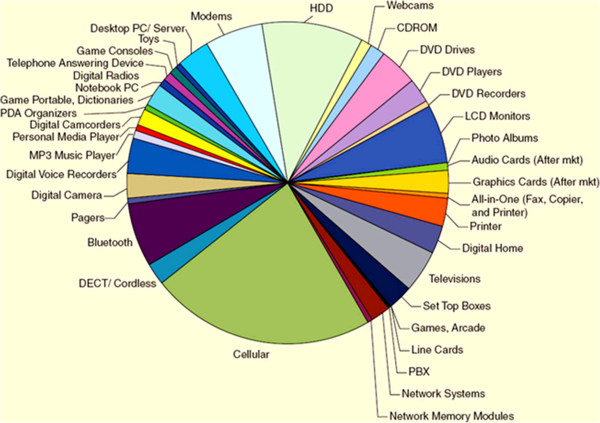
**Various NVSM applications in the electronics industry by market size in 2010.** Reprinted from ref. [22].

The nonvolatile memories offer the system a different opportunity and cover a wide range of applications, from consumer and automotive to computer and communication. Figure 3 shows NVSM memory consumption by various applications in the electronics industry by market in 2010 extending upwards from computers and communication to consumer products [22]. It is noticed that there is a faster growth rate of the digital cellular phone since 1990; the volume of production has increased by 300 times, e.g., from 5 million units per year to about 1.5 billion units per year. Nowadays, flexibility and transparency are particularly of great significance for future electronic applications such as affordable and wearable electronics. Many advanced research technologies are applied to flexible technology to be used in a real electronics area [35]. Although silicon-based semiconductor memories have played significant roles in memory storage applications and communication in consumer electronics, now, the recent focus is turning from rigid silicon-based memory technology into a soft nonvolatile memory technology for low-cost, large-area, and low-power flexible electronic applications. Further, the memory market for the long term is continuously growing, even if with some ups and downs, and this is expected to continue in the coming years [36]. Since innovation drives the semiconductor industry, a new trend with transparency as well as flexibility and 3-D technologies will be attractive and move towards continuous growth in the near future.

**Figure 4:**
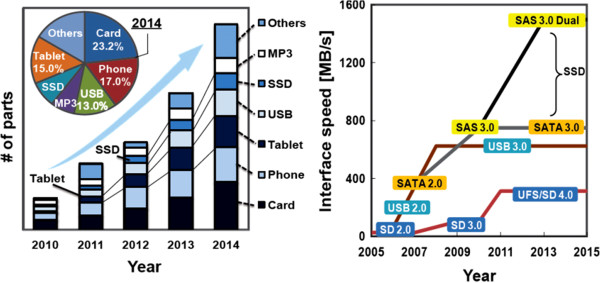
**Growth of NAND Flash market up to 2014 (iSuppli) and the interface speed of various NAND applications.** Reproduced from ref. [37].

Successive creation of new mobile devices leads to the continual growth of NAND products as shown in Figure 4. To meet this market demand, early this year, 30-nm node technologies are in ramping-up phase, 20-nm node technologies are in the phase of transition to mass production, and a 10-nm node technology is under development. In addition, the future market requires high-speed operation even up to approximately 1,500 MB/s in order to satisfy a large amount of data correspondence [37]. However, high-speed operations cause high power consumption and chip temperature increase, which can deteriorate NAND reliability. Hence, reduction of operating voltage is inevitable to achieve the future NAND. Opportunities for the use of 3-D as well as polymer memory design in modern electronic circuits are rapidly expanding, based on the very high performance and unique functionality. However, their practical implementation in electronic applications will ultimately be decided by the ability to produce devices and circuits at a cost that is significantly below that needed to manufacture conventional electronic circuits based on, for example, silicon. If successful, these low-cost fabrication processes will ultimately result in the printing of large-area organic electronic circuits on a sheet of plastic paper using a roll-to-roll method, where low-temperature deposition of organics is followed by metal deposition and patterning in a continuous, high-speed process analogous, perhaps, to processes used in the printing of documents or fabrics.

**Figure 5:**
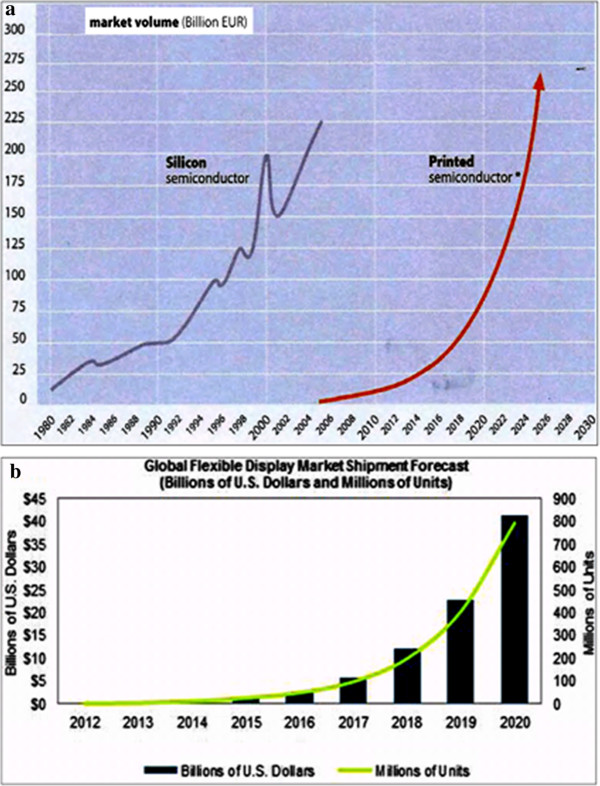
**Market volume (a) and global flexible display market shipment forecast (b).** Reproduced from refs. [38,39].

In recent years, IDTechEx finds that the total market for printed, flexible, and organic electronics will grow from $16.04 billion in 2013 to $76.79 billion in 2023 and this growing trend is expected to continue in the coming years (see Figure 5a). The majority of that is OLEDs (only organic, not printed) and conductive ink used for a wide range of applications. On the other hand, stretchable electronics, logic and memory, and thin-film sensors are much smaller ingredients but having huge growth potential as they emerge from R&D [38]. The report specifically addresses the big picture that over 3,000 organizations are pursuing printed, organic, flexible electronics, including printing, electronics, materials, and packaging companies. While some of these technologies are in use now - indeed there are main sectors of business which have created billion-dollar markets - others are commercially embryonic.

Another key potential market for printed/flexible electronics is next-generation transparent conductive film to replace brittle and expensive indium tin oxide (ITO) in touch screens and displays, lighting, and photovoltaics. Touch display research says that the market for non-ITO transparent conductors will be about $206 million this year and grow to some $4 billion by 2020 as shown in Figure 5b. ‘High demand for touchscreens for notebook and PC size displays has created a shortage of ITO touch sensors since the end of last year to drive more interest in these technologies, and the more flexible and potentially cheaper replacement technologies are getting more mature, notes Jennifer Colegrove, president and analyst, who will speak at the FlexTech workshop on transparent conductors. She notes that Atmel, Fujifilm, Unipixel and Cambrios are all in some phase of production’ [39]. A large amount of the semiconductor market (approximately 20%) is given by the semiconductor memories; thus, the market for chips will develop in the next few years. This study reports that there is an analysis of the production process and the subsequent value chain, which comprises a benchmark analysis of the main segments of the semiconductor industry.

Recently, the 3-D nonvolatile memory structure has also attracted considerable attention due to its potential to replace conventional Flash memory in next-generation NVM applications [37,40]. 3-D memories are gathering increasing attention as future ultra-high-density memory technologies to keep a trend of increasing bit density and reducing bit cost. The NAND Flash market is continuously growing by the successive introduction of innovative devices and applications. To meet the market trend, 3-D NVMs are expected to replace the planar one, especially for 10-nm nodes and beyond. Therefore, the fundamentals and current status of the 3-D NAND Flash memory are reviewed and future directions are discussed [41]. 3-D integration promises to be an excellent replacement of current technologies for the development of NAND Flash memory. Time is running out for planar NAND technology. It will not be long that planar NAND will be completely replaced by 3-D NAND. 3-D NAND promises to satisfy the growing need of NAND memory [37].

Finally, NVM technologies have a bright future since every end-use application needs to store some parameters or some amount of an application program in the on-board NVM to enable it to function. The upcoming NVMs are the big hope for a semiconductor memory market, which provides memories for systems to run with flexibility, reliability, high performance, and low power consumption in a tiny footprint in nearly every electronic application. Recent market trends have indicated that commercialized or near-commercialized circuits are optimized across speed, density, power efficiency, and manufacturability. Flash memory is not suited to all applications, having its own problems with random-access time, bit alterability, and write cycles. With the increasing need to lower power consumption with zero-power standby systems, observers are predicting that the time has come for alternative technologies to capture at least some share in specific markets such as automotive smart airbags, high-end mobile phones, and RFID tags. An embedded nonvolatile memory with superior performance to Flash could see widespread adoption in system-on-chip (SoC) applications such as smart cards and microcontrollers.

### Emerging NVM technologies for applications

**Figure 6:**
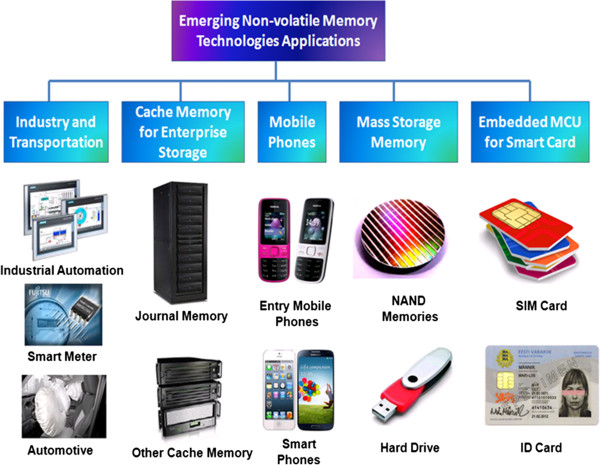
Emerging NVM applications in various markets.

The new emerging nonvolatile random-access memory products address the urgent need in some specific and small-form devices. Therefore, *iRAP* felt a need to do a detailed technology update and market analysis in this industry [42]. Recently, Yole Développement reports describe that emerging memory technologies have great potential to improve future memory devices to be increasingly used in various markets of industry and transportation, enterprise storage, mobile phones, mass storage, and smart cards [43]. Emerging NVM applications in various markets are shown in Figure 6. But there are numerous opportunities existing for novel architectures and applications that these emerging memory technologies can enable. These new emerging NVM products address the urgent need in some specific and small-form devices. Therefore, emerging nonvolatile memory products provide market data about the size of growth of the application segments and the developments of business opportunities. Until now, only FeRAM, PCM, and MRAM were industrially produced and available in low-density chips to only a few players. Thus, the market was quite limited and considerably smaller than the volatile DRAM- and nonvolatile Flash NAND-dominant markets (which enjoyed combined revenues of $50+ billion in 2012). However, in the next 5 years, the scalability and chip density of those memories will be greatly improved and will spark many new applications with NVM market drivers explained in more detail.

**Figure 7:**
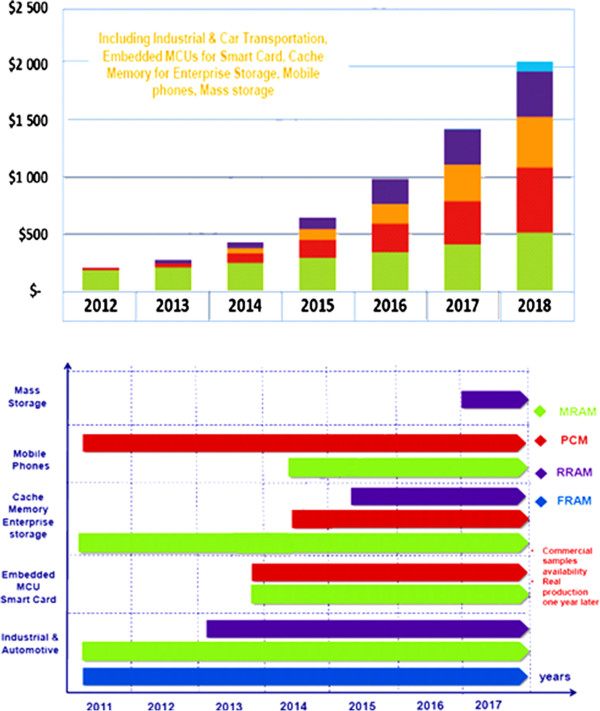
**Emerging NVM market forecast by applications from 2012 to 2018 (in M$).** Reproduced from ref. [43].

Accompanied by the adoption of STT-MRAM and PCM cache memory, enterprise storage will be the largest emerging NVM market. NVM will greatly improve the input/output performance of enterprise storage systems whose requirements will intensify with the growing need for web-based data supported by floating mass servers. In addition, mobile phones will increase their adoption of PCM as a substitute to Flash NOR memory in MCP packages to 1-gigabyte (GB) chips made available by Micron in 2012. Higher-density chips, expected in 2015, will allow access to smart phone applications that are quickly replacing entry-level phones. STT-MRAM is expected to replace SRAM in SoC applications, thanks to lower power consumption and better scalability. Smart cards and microcontrollers (MCU) will likely adopt MRAM/STT-MRAM and PCM as a substitute to embed Flash. Indeed, Flash memory cell size reduction is limited in the future. The NVM could reduce the cell size by 50% and thus be more cost-competitive. Additional features like increased security, lower power consumption, and higher endurance are also appealing NVM attributes. The mass storage markets served by Flash NAND could begin using 3-D RRAM in 2017 to 2018, when 3-D NAND will slow down its scalability as predicted by all of the main memory players. If this happens, then a massive RRAM ramp-up will commence in the next decade that will replace NAND; conditional 3-D RRAM cost-competitiveness and chip density are available. It is expected surely that the emerging NVM business will be very dynamic over the next 5 years, thanks to improvements in scalability/cost and density of emerging NVM chips [44].According to a recently published report from Yole Développement, Emerging Non-volatile Memory Technologies, Industry Trends and Market Analysis, the global market for emerging nonvolatile random-access memory products was projected to have reached $200 million in 2012. This market is expected to increase to $2,500 million by 2018 at an average annual growth at a CAGR of +46% through the forecast period with mobile phones, smart cards, and enterprise storage as main growth drivers (Figure 7). Market adoption of memory is strongly dependent on its scalability. This Yole Développement report provides a precise memory roadmap in terms of technological nodes, cell size, and chip density for each emerging NVM such as FeRAM, MRAM/STT-MRAM, PCM, and RRAM. A market forecast is provided for each technology by application, units, revenues, and also market growth as given a detailed account of emerging NVM market forecast (Figure 7). PCM devices, the densest NVM in 2012 at 1 GB, will reach 8 GB by 2018, which are expected to replace NOR Flash memory in mobile phones and will also be used as a storage class memory in enterprise storage. MRAM/STT-MRAM chips will reach 8 to 16 GB in 2018. They will be widely sold as a storage class memory and possibly as a DRAM successor in enterprise storage after 2018. By 2018, MRAM/STT-MRAM and PCM will surely be the top two NVM on the market. Combined, they will represent a $1.6 billion business by 2018, and their sales will almost double each year, with double-density chips launched every 2 years. FeRAM will be more stable in terms of scalability, with 8- to 16-MB chips available by 2018; the development of a new FRAM material could raise scalability, but we do not expect it to be widely industrialized and commercialized before 2018. FeRAM will grow at a steady growth rate (10% per year) and will focus on industrial and transportation applications because of the low-density availability, whereas RRAM revenues would not really surge by 2018, with the availability of high-density chips of several tens of gigabytes that could replace NAND technology. Meanwhile, it has also been considered by memory technologist experts that for large-volume markets like mass storage NAND, only one technology will be adopted in order to reduce production cost and RRAM seems to be the best candidate. But the real massive adoption of emerging NVM as a replacement for NAND and DRAM will happen after 2020.

### Advances in Flash memory technologies

Flash memory is basically a MOSFET nonvolatile device that can be electrically erased and reprogrammed [3,45]. It is a technology that is primarily used in memory cards and Flash drives for general storage and transfer of data between computers and other digital products. Since the invention of the transistor, NVSM had been the most important invention in the electron device field. The floating gate memory was used to store the information and a tunneling current for programming and erasing operations. The charge is injected into or removed from the floating gate and the floating gate remains in that state, even after power is removed, which means that Flash memory is nonvolatile. The invention of NVSM further gave rise to a new class of memory devices and hence broadened its applications to become ubiquitous. There are a large number of products in the market now which use Flash devices exclusively as secondary storage. Few examples of their applications include medical diagnostic systems, notebook computers, digital audio players, digital cameras, mobile phones, personal digital assistants, digital televisions, universal serial bus (USB) Flash personal disks, Global Positioning Systems, and many more. Semiconductor storage devices store data in tiny memory cells made of very small transistors and capacitors made of semiconductor materials such as silicon. Each cell can hold 1 bit of information and an array of cells stores a large chunk of information. Flash devices are gaining popularity over conventional secondary storage devices like hard disks. The Flash memory fabrication process is compatible with the current CMOS process and is a suitable solution for embedded memory applications. A Flash memory cell is simply a MOSFET cell, except that a polysilicon floating gate [46] (or a silicon nitride charge trap layer) is sandwiched between a tunnel oxide and an inter-polyoxide to form a charge storage layer [47]. Although Flash memory is likely the standard charge storage device for the next generation, scaling may eventually be limited by the tunnel oxide limit [8]. In terms of the operation speed of program and erase, Flash memory requires a thin tunnel oxide to enhance the carrier transport between the floating gate and the silicon substrate. However, the very thin tunnel oxide suffers from many reliability issues like reduction in operation voltage, and after a considerable number of program and erase cycles, the tunnel oxide undergoes deterioration loss [48]. Thus, researchers have focused on possible solutions and proposed alternate technologies, including nitride-based memory, nanocrystal memory, and switching memory. All other nonvolatile memories require integration of new materials that are not as compatible as the conventional CMOS process.

#### ***NOR and NAND Flash memory technologies***

**Figure 8:**
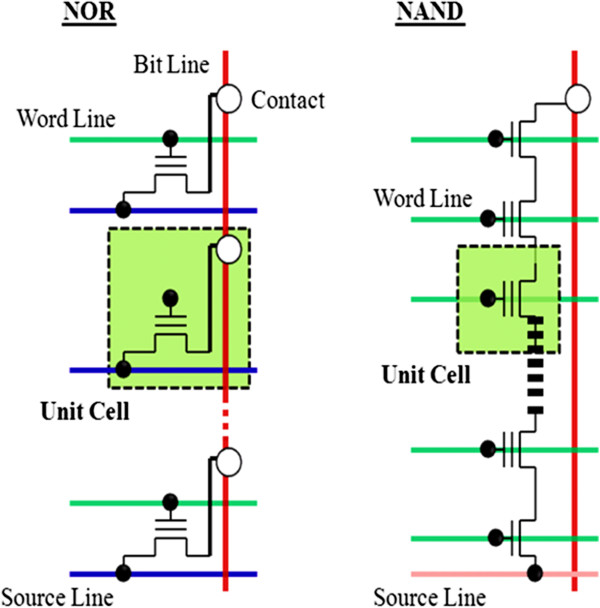
Comparison of NOR Flash array and NAND Flash array architectures.

NOR and NAND Flash, two major Flash types, are dominant in the memory market. NOR Flash has lower density but a random-access interface, while NAND Flash has higher density and interface access through a command sequence [49]. Their corresponding structures are shown in Figure 8. NOR and NAND Flash come from the structure used for the interconnections between memory cells. Intel is the first company to introduce a commercial (NOR type) Flash chip in 1988, and Toshiba released the world's first NAND Flash in 1989 [50]. Depending on how the cells are organized in the matrix, it is possible to distinguish between NAND Flash memories and NOR Flash memories. In NOR Flash, cells are connected in parallel to the bit lines, which notably allow the cells to be read and programmed individually. The parallel connection of NOR Flash cells resemble the parallel connection of transistors in a CMOS NOR gate architecture. On the other hand, in NAND Flash, the cells are connected in series, resembling a NAND gate. The series connections consume less space than the parallel ones, reducing the cost of NAND Flash. It does not, by itself, prevent NAND cells from being read and programmed individually. Most of the engineers and scientists are not so familiar with the differences between these two technologies. Generally, they usually refer to the NOR architecture as ‘Flash’ and are unaware of the NAND Flash technology and its many benefits over NOR [51]. This could be due to the fact that most Flash devices are used to store and run codes (usually small), for which NOR Flash is the default choice, although we are providing some major differences between NOR and NAND Flash technologies by their architecture and the internal characteristic features of the individual Flash.

NOR Flash is slower in erase operation and write operation compared to NAND Flash [52]. This means that NAND Flash has faster erase and write times. Moreover, NAND Flash has smaller erase units, so fewer erases are needed. NOR Flash can read data slightly faster than NAND Flash. NOR Flash offers complete address and data buses to randomly access any of its memory locations (addressable to every byte). This makes it a suitable replacement for older ROM BIOS/firmware chips, which rarely needs to be updated. Its endurance is 10,000 to 1,000,000 erase cycles. NOR Flash is highly suitable for storing codes in embedded systems. Most of today's microcontrollers come with built-in Flash memory [53].

NAND Flash occupies a smaller chip area per cell. This makes NAND Flash available in greater storage densities and at lower costs per bit than NOR Flash. It also has up to ten times the endurance of NOR Flash. NAND is more fit as storage media for large files including video and audio. USB thumb drives, SD cards, and MMC cards are of NAND type [54]. NAND's advantages are fast write (program) and erase operations, while NOR's advantages are random access and byte write capability. NOR's random access ability allows for execute in place (XiP) capability, which is often a requirement in embedded applications. NAND is slow random accessible, while NOR is hampered by having slow write and erase performance. NAND is better suited for filing applications. However, more processors include a direct NAND interface and can boot directly from NAND (without NOR). However, NAND cannot perform read and write operations simultaneously; it can accomplish these at a system level using a method called shadowing, which has been used on PCs for years by loading the BIOS from the slower ROM into the high-speed RAM.

Table 1 highlights the major differences between NOR and NAND. It shows that NAND is ideal for high-capacity data storage while NOR is best used for code storage and execution, usually in small capacities. There are many other differences between these two technologies which will be further discussed individually. However, those listed in the table are enough to strongly differentiate the types of applications using them: NOR is typically used for code storage and execution. This, mainly in capacities up to 4 MB, is common in applications such as simple consumer appliances, low-end cell phones, and embedded applications, while raw NAND is used for data storage in applications such as MP3 players, digital cameras, and memory cards [55-57]. The codes for raw NAND-based applications are stored in NOR devices.

**Table 1:** **Comparison between NOR and NAND Flash memories **[55-57]

**Features**	**NOR**	**NAND**
Memory size	≤512 Mbit	1 to 8 Gbit
Sector size	Approximately 1 Mbit	Approximately 1 Mbit
Program time	9 μs/word	400 μs/page
Erase time	1 s/sector	1 ms/sector
Read access time	<80 ns	20 μs
Write parallelism	8 to 16 words	2 Kbyte
Output parallelism	Byte/word/dword	Byte/word
Read parallelism	8 to 16 words	2 Kbyte
Access method	Random	Sequential
Price	High	Very low
Reliability	Standard	Low

#### ***Scaling and challenges of Flash memory technologies***

**Figure 9:**
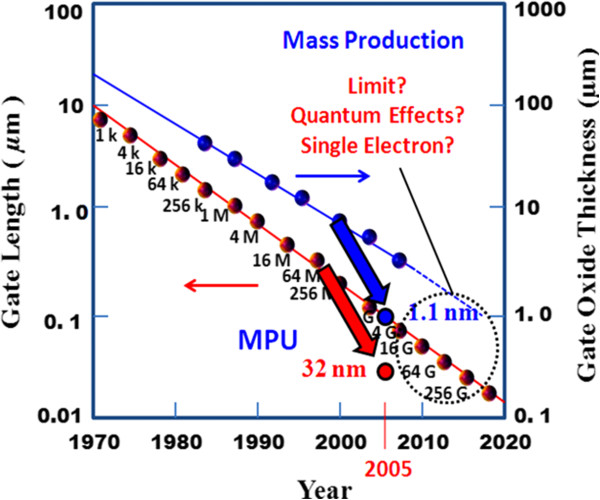
**The trend of MOSFET scaling from ITRS.** Reproduced from ITRS Corp.

Currently, there have been increasing demands on reducing the feature size in microelectronic products and more interest in the development of Flash memory devices to meet the growing worldwide demand. A conventional FG memory device must have a tunnel oxide layer thickness of 8 nm to prevent charge loss and to make 10 years' data retention certain. This necessity will limit scalability for Flash memory devices [8,58]. Thus, in order to meet technology scaling in the field of memory and data storage devices, mainstream transistor-based Flash technologies will be developed gradually to incorporate material and structural innovations [59]. Dielectric scaling in nonvolatile memories has been reached near to the point where new approaches will be required to meet the scaling requirements while simultaneously meeting the reliability and performance requirements for future products. High-dielectric-constant materials are being explored as possible candidates to replace both the traditional SiO_2_ and oxide/nitride/oxide (ONO) films used in Flash memory cells. Flash cell scaling has been demonstrated to be really possible and to be able to follow Moore's law down to the 90-nm technology generations. The technology development and the consolidated know-how are expected to sustain the scaling trend down to the 50-nm technology node and below as forecasted by the International Technology Roadmap for Semiconductors (ITRS) in Figure 9, which indicates that the silicon MOSFET was already in the nanoscale. The minimum feature size of an individual CMOSFET has shrunk to 15 nm with an equivalent gate oxide thickness (EOT) of 0.8 nm in 2001 [13]. However, semiconductor Flash memory scaling is far behind CMOS logic device scaling. For example, the EOT of the gate stack in semiconductor Flash memory is still more than 10 nm. Moreover, semiconductor Flash memory still requires operation voltages of more than 10 V, which is still far from the operation voltage of CMOS logic devices. It is important to scale the EOT of the gate stack to achieve a small memory cell size and also prolong battery life.

**Figure 10:**
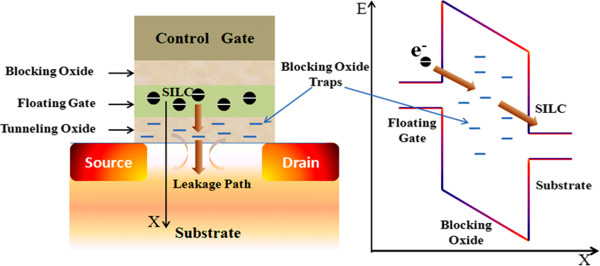
**Schematic plots of a Flash memory cell and the degradation of its tunnel oxide.** The degradation leads to the formation of percolation paths responsible for the FG charge loss, hence the loss of the stored information. The presence of traps in the energy barrier yields the trap-assisted tunneling mechanism and originates the stress-induced leakage current (SILC).

Another limitation of FG technology is that tunnel oxide scaling is limited by stress-induced leakage current (SILC) related to charge transfer problem as indicated in Figure 10[60,61]. The SILK increases with decreasing oxide thickness. This can be attributed to tunneling assisted by the traps in the bulk of the dielectric. Trap-assisted tunneling can take place at very low electric fields. If the density of traps is increased, the leakage will also increase. Electrical stress can increase the number of these traps. So it becomes an important limitation of scaling down the memory device [62]. For EOT < 8 nm, a single oxide trap will cause to complete the charge loss in the FG Flash cell. The scaling of the gate stacks and operation voltages are often related to each other. A tunnel oxide thickness of more than 8 nm is currently used in the commercial Flash memory chip to meet the 10 years' data retention time requirement. If the tunnel oxide were to be scaled below 2 nm, the operation voltage could be reduced from more than 10 V to below 4 V [63]. Unfortunately, the retention time would also be reduced, from 10 years to several seconds. This physical damage to the tunnel oxide during the cycling process causes data retention problems, program disturbance, read disturbance, and erratic characteristic behavior of the FG memory cell. Such problems severely limit the reliability and multilevel cell operation. This basic limitation of the tunnel oxide thickness becomes increasingly important with scaling. New storage node concepts are also becoming attractive as an alternative approach to address some of the dielectric scaling limitations. Flash memory adopts a charge stored in a silicon nitride as the trapping layer, which exhibits significantly reduced defect-related leakage current and very low SILC as compared to SiO_2_ with a similar EOT [64]. Such a relentless reduction of device dimensions has many challenges like retention, endurance, reduction in the number of electrons in the FG, dielectric leakage, cell-to-cell cross talk, threshold voltage shift, and reduction in memory window margins [65,66]. The key concept of real scaling issues such as material and structural changes in Flash memory technologies is provided in detail in the next distinct part.

#### ***FG Flash memory technology***

**Figure 11:**
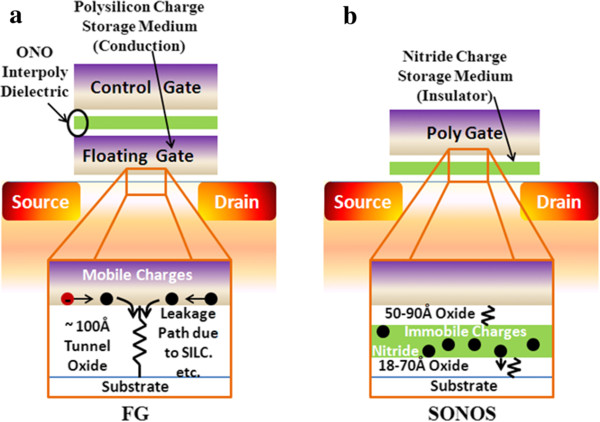
**Schematics of the conventional FG memory and SONOS.** Schematics of **(a)** floating gate and thin-film storage-based embedded nonvolatile memory bit cells, depending on the charge stored inside the gate dielectric of a MOSFET, and **(b)** the nitride traps (SONOS), embedded into the gate oxide of a MOSFET.

The FGNV memory is a basic building block of Flash memory, which is based on FG thin-film storage (TFS) memories that have been developed with the addition of an erase gate configuration. The conventional FG memory (Figure 11a) consists of a MOSFET configuration that is modified to include polysilicon as a charge storage layer surrounded by an insulated inner gate (floating gate) and an external gate (control gate). This what makes Flash memory nonvolatile and all floating gate memories to have the same generic cell structure. Charge is transferred to or from the floating gate through a thin (8 to 10 nm) oxide [1,67]. Because the floating gate is electrically isolated by the oxide layer, any electrons placed on it are trapped there. Flash memory works by adding (charging) or removing (discharging) electrons to and from a floating gate. A bit's 0 or 1 state depends upon whether or not the floating gate is charged or discharged. When electrons are present on the floating gate, current cannot flow through the transistor and the bit state is ‘0’. This is the normal state for a floating gate. When electrons are removed from the floating gate, current is allowed to flow and the bit state is ‘1’. The FG memory has achieved high density, good program/erase speed, good reliability, and low operating voltage and promotes endurance for Flash memory application.

#### ***SONOS memory technology***

In order to solve the scaling issue of the FG memory, the SONOS memory has been proposed as a Flash technology since the 1980s [68,69]. The acronym SONOS is derived from the structure of the device as shown in Figure 11b. The SONOS device is basically a MOSFET, where the gate has been replaced by an ONO dielectric. The SONOS memory has a better charge retention than the FG memory when the FG bit cell's tunneling oxide layer is below 10 nm [70]. Moreover, the SONOS memory exhibits many advantages, e.g., easy to fabricate, high program/erase (P/E) speed, low programming voltage and power consumption, and better potential for scalability below the 70-nm node, according to the ITRS [71]. The charge, holes or electrons, are injected into the nitride layer using direct tunneling through the tunnel oxide layer. The nitride layer is electrically isolated from the surrounding transistor, although charges stored on the nitride directly affect the conductivity of the underlying transistor channel. Since the SONOS memory possesses spatially isolated deep-level traps, a single defect in the tunneling oxide will not cause discharge of the memory cell. The thickness of the top oxide is important to prevent the Fowler-Nordheim tunneling of electrons from the gate during erase. When the polysilicon control gate is biased positively, electrons from the transistor source and drain regions tunnel through the oxide layer and get trapped in the silicon nitride. This results in an energy barrier between the drain and the source, raising the threshold voltage *V*_th_ (the gate-source voltage necessary for current to flow through the transistor). Moreover, the nitride layer is electrically isolated from the surrounding transistor, although charges stored on the nitride directly affect the conductivity of the underlying transistor channel. The oxide/nitride sandwich typically consists of a 2-nm-thick oxide lower layer, a 5-nm-thick silicon nitride middle layer, and a 5- to 10-nm-thick oxide upper layer [72,73]. However, SONOS-type Flash memories have several drawbacks such as shallow trap energy level, erase saturation, and vertical stored charge migration [74]. The programming speed and operating voltage problems can be solved by reducing the tunnel oxide thickness. At low tunnel oxide thickness, the issues that impact SONOS-type memories include erase saturation and vertical charge migration, which seriously degrade the retention capability of the memory [75]. Thus, many concerns still remain for the SONOS type of memories, which will be discussed in the next section.

#### ***Limitations of FG and SONOS memory technologies***

Scaling demands very thin gate insulators in order to keep short channel effects and control the shrinkage of the device size and maximize the performance. When the tunneling oxide thickness is below 10 nm, the storaged charge in the FG is easy to leak due to a defect in the tunneling oxide formed by repeated write/erase cycles or direct tunneling current.

**Figure 12:**
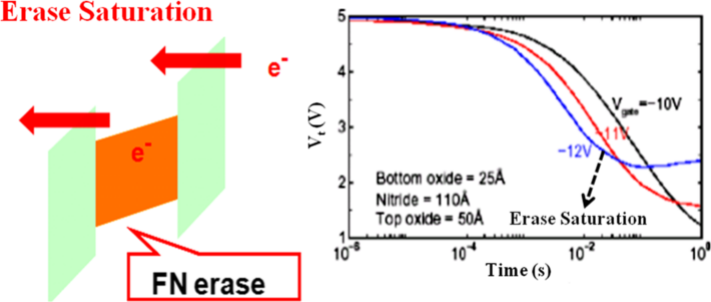
**Fowler-Nordheim (FN) tunneling of electrons from the gate during erase and erase saturation in SONOS nonvolatile memory.** This indicates the reduced memory window as the erase voltage is increased. Reproduced from ref. [74].

The tunneling gate oxide thickness in a conventional Flash memory cannot be scaled down to sub-7 nm because of charge retention [76]. The SONOS Flash memory can relieve the problem but still has a relatively thick gate dielectric thickness of about 7 nm. Therefore, conventional SONOS Flash memory also has a scaling-down problem. Many studies have shown that the charge retention characteristics in scaled SONOS nonvolatile memory devices with a low gate oxide thickness and at high temperature are problematic with shallow-level traps [48,77,78]. For the conventional SONOS memory, erase saturation and vertical stored charge migration [79,80] are the two major drawbacks; the most challenging tasks are how to maintain an acceptable charge capability of the discrete storage nodes and how to fabricate nanocrystals with constant size, high density, and uniform distributions [81]. When the trap energy level is shallow, erase saturation and vertical migration occur and the electron charge decay rate increases due to low tunnel oxide thickness, issues that impact SONOS-type memories as shown in Figure 12. This erase saturation makes SONOS erase less as the erase voltage or the tunnel oxide thickness is increased. Since the SONOS memory uses silicon nitride as a charge trapping layer, the electrons in the Si sub-conduction band will tunnel through the tunneling oxide and a portion of the nitride, and this consequently degrades the program speed. Besides this, the conduction band offset of nitride is only 1.05 eV and back-tunneling of the trapped electron may also occur. Although applying a very high electric field may accelerate the de-trapping rate, the gate electron injection current exceeds the de-trapping but resulting in practically an increase in charge and no erasing. Using an ultra-thin (<2 nm) tunnel oxide offers an efficient charge direct tunneling erase and opens a memory window. However, the direct tunneling cannot be turned off at a low electric field, leading to poor retention and read disturb. Thus, the SONOS memory cannot be used for NAND Flash without further innovation of new memory technologies. The main reason for the growth of emerging NVM technologies is that scaling has now become a serious issue for the memory industry. Not only are many of these new technologies inherently more scalable, but also they seem well suited to the next generation of mobile computing and communications that will demand high-capacity memories capable of storing and rapidly accessing video and a large database without overburdening battery power sources.

Many alternate device structures are proposed to hopefully circumvent these scaling challenges and to improve the device performance. In an effort to continue Moore's law and overcome the ultimate limitations of MOS-based memory devices, other storage concepts have been proposed in search of the ‘unified memory’. The ideal memory device or the so-called ‘unified memory’ would satisfy simultaneously three requirements: high speed, high density, and nonvolatility. At the present time, such an ideal memory has not been developed. FGNVSM has high density and nonvolatility, but its P/E speed is low. DRAM has high speed (approximately 10 ns) and relatively high density, but it is volatile. SRAM has very high speed (approximately 5 ns), but it suffers from very low density and volatility. Many nonvolatile memory devices have been proposed on the basis of changing charge storage materials and new device concepts for the ‘unified memory’. These structures will be considered in the next sections. In light of such issues, emerging memory solutions seem to be a key technology.

### Current emerging memory technologies

Recent studies have revealed that there is a close correlation among existing and emerging memory technologies in view of scalability. The scaling trend of memory transition leads to smaller and smaller memory devices, which have been routinely observed. To further support this assertion, another set of current progress in memory technology is described to the increasing importance of memory to users' experience and the importance of memory to system performance. There are many emerging memory technologies which are trying to replace existing memory technologies in the market. These new memory devices such as RRAM, PCM, and STT-RAM have read/write/retention/endurance characteristics different from those of conventional SRAM, DRAM, and Flash [82]. But the ideal characteristics of new emerging memory technologies have to be meeting the performance of SRAM and the density of NAND Flash in terms of stability, scalability, and switching speed. Thus, going beyond the traditional bistable memory, the possibilities of multilevel, high-performance memory devices suitable for market must be explored. Currently, there are several technologies that show some promise; some of these new emerging technologies are MRAM, FeRAM, PCM, STT-RAM, nano-random-access memory (NRAM), racetrack memory, RRAM and memristor, molecular memory, and many others [10,83]. Each of these memory technologies will be briefly outlined and discussed in the following sections. In view of the commercial production, currently, MRAM, FeRAM, and PCM are in commercial production but still remain limited to niche applications relative to DRAM and NAND Flash. There is a prospect that among the emerging memory technologies, MRAM, STT-RAM, and RRAM are the most promising ones, but they are still many years away from competing for industry adoption [84]. It is necessary for any new technology to be able to deliver most for industry adoption. For industry adoption on a mass scale, some parameters must be matched with existing memory technologies. In consideration of new technology for industry application, the scalability of the technology, speed of the device, power consumption to be better than existing memories, endurance, densities, better than existing technologies and most importantly the cost; if the emerging technology can only run one or two of these attributes, then, at most desirable, it is likely to be resigned to niche applications.

#### ***MRAM***

**Figure 13:**
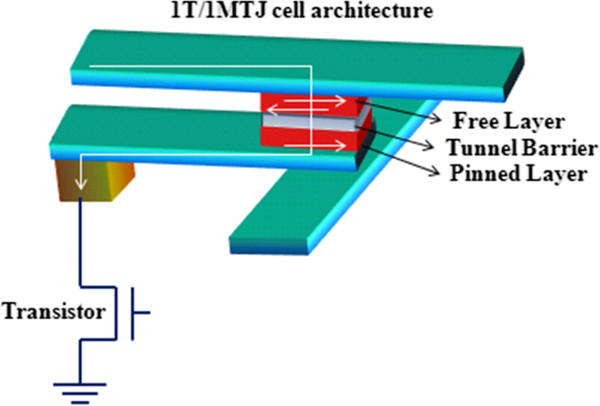
Basic MRAM cell structure.

MRAM or magnetic RAM is a nonvolatile RAM technology under development since the 1990s. RRAM methods of storing data bits use magnetic charges instead of the electrical charges used by DRAM and SRAM technologies. MRAM, first developed by IBM in the 1970s [85], is expected to replace DRAM as the memory standard in electronics. MRAM is basically based on memory cells having two magnetic storage elements, one with a fixed magnetic polarity and another with a switchable polarity. These magnetic elements are positioned on top of each other but separated by a thin insulating tunnel barrier as shown in the cell structure in Figure 13. Moreover, scientists define a metal as magnetoresistive if it shows a slight change in electrical resistance when placed in a magnetic field. By combining the high speed of static RAM and the high density of DRAM, proponents say that MRAM could be used to significantly improve electronic products by storing greater amounts of data, enabling it to be accessed faster while consuming less battery power than existing electronic memories. Technically, it works with the state of the cell, which is sensed by measuring the electrical resistance while passing a current through the cell. Because of the magnetic tunnel effect [86], if both magnetic moments are parallel to each other, then the electrons will be able to tunnel and the cell is in the low resistance ‘ON’ state. However, if the magnetic moments are antiparallel, the cell resistance will be high. The memory characteristics of MRAM of writing and erasing are fulfilled by passing a current through the write line to induce a magnetic field across the cell. MRAM has been slowly getting off the ground but has now entered the market and will become increasingly available for mass production in the couple of years and beyond. Currently, it has reached some level of commercial success in niche applications [87]. Various companies such as Samsung, IBM, Hitachi and Toshiba, and TSMC are actively developing variant technologies of MRAM chips. In view of power consumption and speed, MRAM competes favorably than other existing memories such as DRAM and Flash, with an access time of a few nanoseconds [88-90]. Although it has some limitation during the ‘write’ operation, the smaller cell size could be limited by the spread of the magnetic field into neighboring cells and need an amendment to compete completely as a universal memory. The price of MRAM is also another issue and considered a limiting factor, with prices far in excess of all the currently established memories at approximately £2 to £3 ($3 to $5) per megabyte [91]. According to this price level, MRAM is in excess of 1,000 times the price of Flash memory and over 10,000 times the price of hard disk drives. It is expected that of the next-generation memory technologies, MRAM, in the future, will have the biggest market, followed by FeRAM, PCRAM, and memristors.

#### ***STT-MRAM***

**Figure 14:**
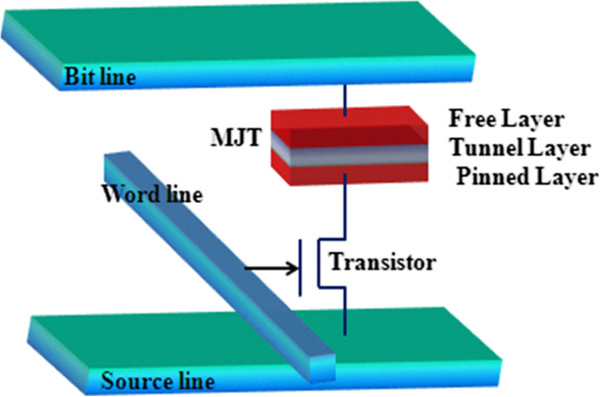
Basic STT-RAM cell structure.

STT-MRAM is a magnetic memory technology that exerts the base platform established by an existing memory called MRAM to enable a scalable nonvolatile memory solution for advanced process nodes [92,93]. It is a new kind of magnetic RAM with the following features: fast read and write times, small cell sizes, potentially even smaller, and compatibility with existing DRAM and SRAM. As we have discussed in the previous section, MRAM stores data according to the magnetization direction of each bit and the nanoscopic magnetic fields set the bits in conventional MRAM. On the other hand, STT-MRAM uses spin-polarized currents, enabling smaller and less energy-consuming bits. The basic cell structure of STT-RAM is depicted in Figure 14. In addition, STT-RAM writing is a technology in which an electric current is polarized by aligning the spin direction of the electrons flowing through a magnetic tunnel junction (MTJ) element. Data writing is performed by using the spin-polarized current to change the magnetic orientation of the information storage layer in the MTJ element [94]. The resultant resistance difference of the MTJ element is used for information readout. STT-RAM is a more appropriate technology for future MRAM produced using ultra-fine processes and can be efficiently embedded in subsequent generations of such semiconductor devices as FPGAs, microprocessors, microcontrollers, and SoC. A special bonus for embedded designers is the fact that the internal voltage STT-RAM requires is only 1.2 V. The difference between STT-MRAM and a conventional MRAM is only in the writing operation mechanism; the read system is the same. The memory cell of STT-MRAM is composed of a transistor, an MTJ, a word line (WL), a bit line (BL), and a source line (SL) [95]. Currently, STT-RAM is being developed in companies including Everspin, Grandis, Hynix, IBM, Samsung, TDK, and Toshiba. However, for STT-RAM to be adopted as a universal mainstream semiconductor memory, some key challenges should be resolved: the simultaneous achievement of low switching current and high thermal stability. It must be dense (approximately 10 F^2^), fast (below 10 ns of read and write speeds), and operating at low power [96].

#### ***FeRAM***

**Figure 15:**
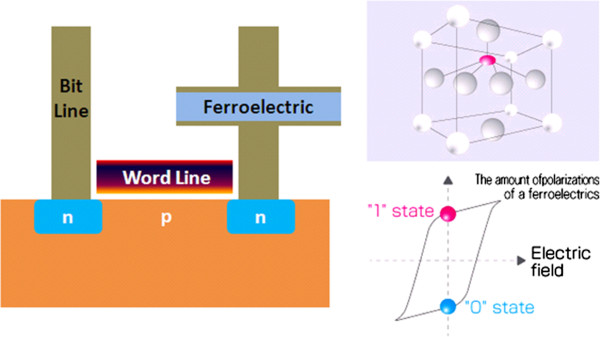
**Basic structure of a FeRAM cell.** The crystal structure of a ferroelectric and an electric polarization-electric field hysteresis curve are also shown.

FeRAM is a nonvolatile RAM that combines the fast read and write access of DRAM cells, consisting of a capacitor and transistor structure as shown in Figure 15. The cell is then accessed via the transistor, which enables the ferroelectric state of the capacitor dielectric to be sensed. In spite of its name, FeRAM does not contain iron. The polarization properties of a ferroelectric substance are used as a memory device. Today's FeRAM uses lead zirconate titanate (PZT); other materials are being considered. The main developer of FeRAM is Ramtron International. FeRAM is the most common kind of personal computer memory with the ability to retain data when power is turned off as do other nonvolatile memory devices such as ROM and Flash memory [97]. In a DRAM cell, the data periodically need refreshing due to the discharging of the capacitor, whereas FeRAM maintains the data without any external power supply. It achieves this by using a ferroelectric material in the place of a conventional dielectric material between the plates of the capacitor. When an electric field is applied across dielectric or ferroelectric materials, it will polarize, and while that field is removed, it will depolarize. But the ferroelectric material exhibits hysteresis in a plot of polarization versus electric field, and it will retain its polarization. One disadvantage of FeRAM is that has a destructive *read* cycle. The read method involves writing a bit to each cell; if the state of the cell changes, then a small current pulse is detected by indicating that the cell was in the OFF state. However, it is a fast memory that can endure a high number of cycles (e.g., 10^14^) [98], meaning that the requirement for a *write* cycle for every *read* cycle will not result in short product lives with a very low power requirement. It is expected to have many applications in small consumer devices such as personal digital assistants (PDAs), handheld phones, power meters, and smart cards, and in security systems. FeRAM is faster than Flash memory. It is also expected to replace EEPROM and SRAM for some applications and to become a key component in future wireless products. Even after FeRAM has achieved a level of commercial success, with the first devices released in 1993 [99,100], current FeRAM chips offer performance that is either comparable to or exceeding current Flash memories [98,101], but still slower than DRAM.

#### ***PCRAM***

**Figure 16:**
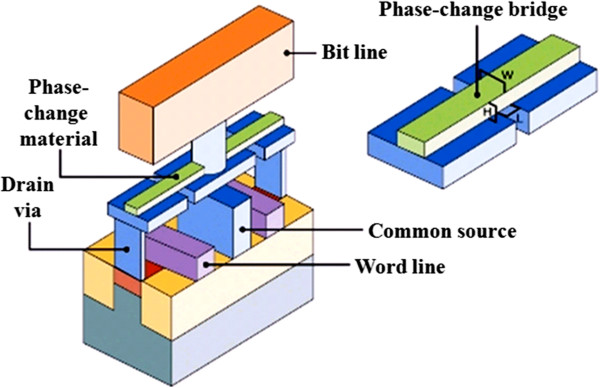
**Basic PCRAM cell structure.** Reproduced from IBM-Macronix-Qimonda.

PCRAM, also known as PCM, perfect RAM (PRAM), OUM, and chalcogenide RAM (CRAM), is a type of nonvolatile RAM based on a class of material called chalcogenide glasses that can exist in two different phase states (e.g., crystalline and amorphous) [102,103]. The basic PCRAM cell structure is depicted in Figure 16. Most phase-change materials contain at least one element from group 6 of the periodic table, and the choice of available materials can be further widened by doping these materials [104-107]. In particular, the most promising are the GeSbTe alloys which follow a pseudobinary composition (between GeTe and Sb_2_Te_3_), referred to as GST. These materials are in fact commonly used as the data layer in rewritable compact disks and digital versatile disks (CD-RW and DVD-RW) where the change in optical properties is exploited to store data. The structure of the material can change rapidly back and forth between amorphous and crystalline on a microscopic scale. The material has low electrical resistance in the crystalline or ordered phase and high electrical resistance in the amorphous or disordered phase. This allows electrical currents to be switched *ON* and *OFF*, representing digital high and low states. This process has been demonstrated to be on the order of a few tens of nanoseconds [108], which potentially makes it compatible with Flash for the *read* operation, but several orders of magnitude faster for the *write* cycle. This makes it possible for PCM to function many times faster than conventional Flash memory while using less power. In addition, PCM technology has the potential to provide inexpensive, high-speed, high-density, high-volume nonvolatile storage on an unprecedented scale. The physical structure is three-dimensional, maximizing the number of transistors that can exist in a chip of fixed size. PCM is sometimes called perfect RAM because data can be overwritten without having to erase it first. Possible problems facing PCRAM concern the high current density needed to erase the memory; however, as cell sizes decrease, the current needed will also decrease. PCM chips are expected to last several times as long as currently available Flash memory chips and may prove cheaper for mass production. Working prototypes of PCM chips have been tested by IBM, Infineon, Samsung, Macronix, and others. Also, the production of PCM has been announced recently by both collaborations between Intel and STMicroelectronics as well as with Samsung [109,110].

#### ***Comparison of primary contenders for MRAM, STT-RAM, FeRAM, and PCM technologies***

Before going to other emerging memories, we herein provide a comparison among MRAM, FeRAM, and PCM. The specific features of these memory devices are provided in Table 2. Relatively mature, new-material memories such as MRAM, STT-RAM, FeRAM, and PCM can offer a variety of features that have potential to be the candidates for next-generation nonvolatile memory devices. Brand-new concepts such as RRAM, molecular, organic/polymer, and other nanowire-based memory technologies have also been proposed. These are discussed in detail in the following section.

**Table 2:** Summary of primary contenders for MRAM, FeRAM, STT-RAM, and PCM technologies

**Features**	**FeRAM**	**MRAM**	**STT-RAM**	**PCM**
Cell size (F^2^)	Large, approximately 40 to 20	Large, approximately 25	Small, approximately 6 to 20	Small, approximately 8
Storage mechanism	Permanent polarization of a ferroelectric material (PZT or SBT)	Permanent magnetization of a ferromagnetic material in a MTJ	Spin-polarized current applies torque on the magnetic moment	Amorphous/polycrystal phases of chalcogenide alloy
Read time (ns)	20 to 80	3 to 20	2 to 20	20 to 50
Write/erase time (ns)	50/50	3 to 20	2 to 20	20/30
Endurance	10^12^	>10^15^	>10^16^	10^12^
Write power	Mid	Mid to high	Low	Low
Nonvolatility	Yes	Yes	Yes	Yes
Maturity	Limited production	Test chips	Test chips	Test chips
Applications	Low density	Low density	High density	High density

#### ***RRAM***

**Figure 17:**
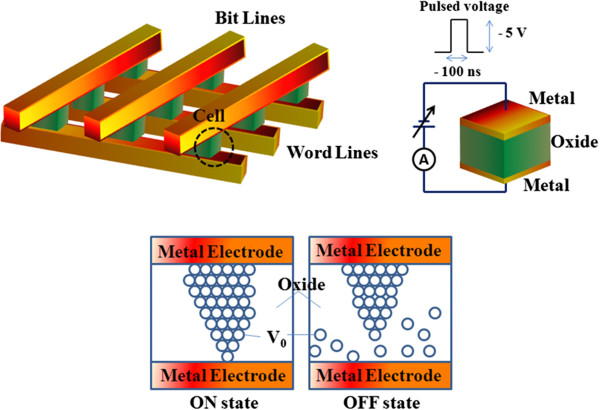
**Basic RRAM cell structure.** A schematic diagram of the mechanism of the resistive switching in a metal/oxide/metal-structured memory cell is also shown. Reproduced from ref. [123].

RRAM is a disruptive technology that can revolutionize the performance of products in many areas, from consumer electronics and personal computers to automotive, medical, military, and space. Among all the current memory technologies, RRAM is attracting much attention since it is compatible with the conventional semiconductor processes. Memristor-based RRAM is one of the most promising emerging memory technologies and has the potential of being a universal memory technology [111]. It offers the potential for a cheap, simple memory that could compete across the whole spectrum of digital memories, from low-cost, low-performance applications up to universal memories capable of replacing all current market-leading technologies, such as hard disk drives, random-access memories, and Flash memories [112]. RRAM is a simple, two-terminal metal-insulator-metal (MIM) bistable device as shown in the basic configuration in Figure 17. It can exist in two distinct conductivity states, with each state being induced by applying different voltages across the device terminals. RRAM uses materials that can be switched between two or more distinct resistance states. Many companies are investing metal oxide nanolayers switched by voltage pulses. Researchers generally think that the pulses' electric fields produce conducting filaments through the insulating oxide. HP Labs plans to release prototype chips this year based on ‘memristors’ in which migrating oxygen atoms change resistance [113]. Xu et al. have also defined that among all the technology candidates, RRAM is considered to be the most promising as it operates faster than PCRAM and it has a simpler and smaller cell structure than magnetic memories (e.g., MRAM or STT-RAM) [114]. In contrast to a conventional MOS-accessed memory cell, a memristor-based RRAM has the potential of forming a cross-point structure without using access devices, achieving an ultra-high density. This device is based on the bistable resistance state found for almost any oxide material, including NiO, ZrO_2_, HfO_2_, SrZrO_3_, and BaTiO_3_[115-119]. Currently, Samsung and IBM are actively investigating RRAM.

Kamiya et al. have revealed by a theoretical mechanism that RRAM shows filamentary-type resistive switching, where the oxygen vacancy is considered to form conductive filaments in the resistive material as shown in Figure 17[120]. The formation and disruption of these filaments are thus the mechanisms responsible for the ON-OFF switching in RRAM devices. The key issue is, therefore, to reveal electronic roles in the formation and disruption of the vacancy filaments. RRAM can be switched between the low resistance state (LRS) and the high resistance state (HRS) of the resistive material by applying voltages to the electrodes. Lee has explained that during the SET process, the current level increases from HRS to LRS as the voltage increases from 0 V to the critical point which is called the set voltage (*V* set), while the current level abruptly decreases from LRS to HRS at the reset voltage (*V* reset) under the RESET process. The SET and RESET processes are repeatedly carried out by sweeping the gate voltage with the binary states LRS and HRS [121]. Wang and Tseng and Lin et al. have indicated that the interface plays an important role in enhancing the performances of RRAM [122,123]. Recently, Goux et al. have explained that using a stacked RRAM structure has been shown to be one of the most promising methods to improve the memory characteristics [124]. Although being a most promising memory element, critical issues for the future development of RRAM devices are reliable, such as data retention and memory endurance [125]. A data retention time of over 10 years can be extrapolated from retention characteristics measured at high temperatures and a memory endurance of over 10^6^ cycles [126]. Therefore, a statistical study of reliability, availability, and maintainability is essential for the future development of RRAM.

#### ***Polymer memory***

**Figure 18:**
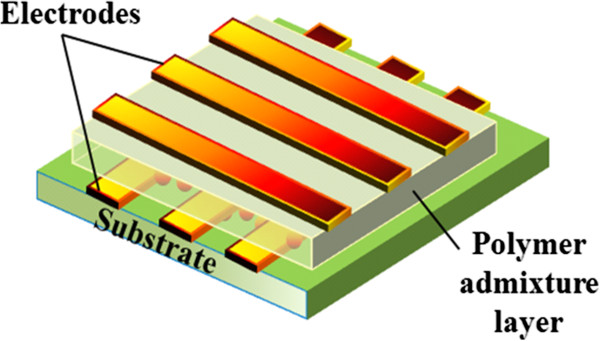
Structure of a polymer memory device.

Throughout the last few years, polymers have found growing interest as a result of the rise of a new class of nonvolatile memories. In a polymer memory, a layer consists of molecules and/or nanoparticles in an organic polymer matrix is sandwiched between an array of top and bottom electrodes as illustrated in Figure 18. Moreover, polymer memory has the advantage of a simple fabrication process and good controllability of materials [127]. Polymer memory could be called digital memory with the latest technology. It is not possible for a silicon-based memory to be established in less space, but it is possible for polymer memory. Ling et al. explained that polymer materials have simplicity in structure, free *read* and *write* capability, better scalability, 3-D stacking ability, low-cost potential, and huge capacity of data storage [128]. They revealed that a polymer memory stores information in a manner that is entirely different from that of silicon-based memory devices. Rather than encoding ‘0’ and ‘1’ from the number of charges stored in a cell, a polymer memory stores data on the basis of high and low conductivity while responding to an applied voltage. Among the large number of emerging memory technologies, polymer memory is the leading technology. It is mainly because of its expansion capability in 3-D space [129] since most polymers are organic materials consisting of long chains of single molecules. Prior to polymer memory fabrication, deposition of an organic layer is usually done by the sol-gel spin coating technique. All the other necessary constituent materials are dissolved in a solvent which is then spin-coated over a substrate. When the solvent is evaporated, a thin film of material with 10- to 100-nm thickness is successfully deposited at bottom electrodes. Top electrodes are deposited as the final step. The conductivity of the organic layer is then changed by applying a voltage across the memory cell, allowing bits of data to be stored in the polymer memory cell. When the polymer memory cell becomes electrically conductive, the electrons are introduced and removed. Even the polymer is considered as a ‘smart’ material to the extent that functionality is built into the material itself of switchability and charge store. This will open up tremendous opportunities in the electronics world, where tailor-made memory materials represent an unknown territory. The nonvolatileness and other features are inbuilt at the molecular level and offers very high advantages in terms of cost. But turning polymer memory into a commercial product would not be easy. Memory technologies compete not only on storage capacity but on speed, energy consumption, and reliability. ‘The difficulty is in meeting all the requirements of current silicon memory chips,’ says Thomas, the Director of Physical Sciences at IBM's Watson Research Center in Yorktown Heights, NY. They are likely to be limited to niche applications [130].

#### ***Racetrack memory***

**Figure 19:**
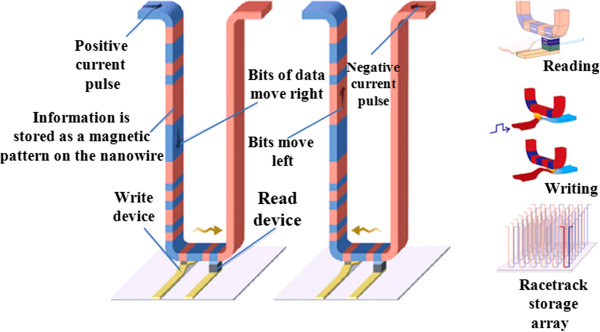
**Racetrack memory diagram showing an array of U-shaped magnetic nanowires.** The nanowires are arranged vertically like trees in a forest and a pair of tiny devices that read and write the data. Adopted from IBM.

In a racetrack memory, information is stored on a U-shaped nanowire as a pattern of magnetic regions with different polarities. The U-shaped magnetic nanowire is an array of keys, which are arranged vertically like trees in a forest as shown in Figure 19. Achieving capacities comparable to vertical RM or hard drives would require stacks of these arrays. The nanowires have regions with different magnetic polarities, and the boundaries between the regions represent 1 or 0 s, depending on the polarities of the regions on either side [131,132]. The magnetic information itself is then pushed along the wire, past the write and read heads by applying voltage pulses to the wire ends. The magnetic pattern to speed along the nanowire, while applying a spin-polarized current, causes the data to be moved in either direction, depending on the direction of the current. A separate nanowire perpendicular to the U-shaped ‘racetrack’ writes data by changing the polarity of the magnetic regions. A second device at the base of the track reads the data. Data can be written and read in less than a nanosecond. A racetrack memory using hundreds of millions of nanowires would have the potential to store vast amounts of data [133,134]. In this way, the memory requires no mechanical moving of parts and it has a greater reliability and higher performance than HDDs, with theoretical nanosecond operating speeds. For a device configuration where data storage wires are fabricated in rows on the substrate, conventional manufacturing techniques are adequate. However, for the maximum possible memory density, the storage wires are proposed to be configured rising from the substrate in a ‘U’ shape, giving rise to a 3-D forest of nanowires. While this layout does allow high data storage densities, it also has the disadvantage of complex fabrication methods, with so far, only 3-bit operation of the devices demonstrated [133]. As the access time of the data is also dependent on the position of the data on the wire, these would also be performance losses if long wires are used to increase the storage density further. The speed of operation of the devices has also been an issue during development, with much slower movement of the magnetic domains than originally predicted. This has been attributed to crystal imperfections in the permalloy wire, which inhibit the movement of the magnetic domains. By eliminating these imperfections, a data movement speed of 110 m/s has been demonstrated [133].

### Other new memory technologies

Researchers are already working hard on several emerging technologies, as discussed in previous sections, to pursue storage-class memories with a more traditional design than that of the racetrack memory, which places the bits in horizontal arrays.

#### ***Molecular memory***

**Figure 20:**
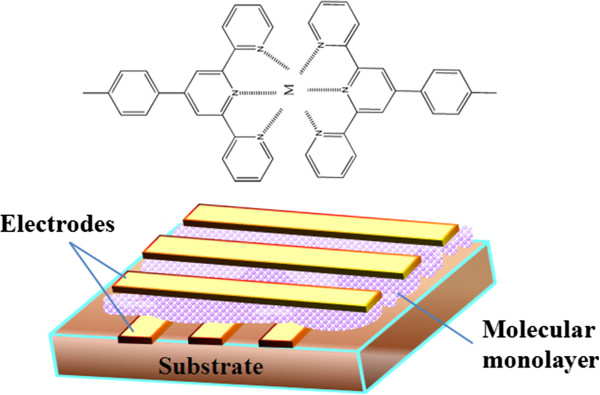
Cell structure of a molecular memory device.

A molecular memory is a nonvolatile data storage memory technology that uses molecular species as the data storage element, rather than, e.g., circuits, magnetics, inorganic materials, or physical shapes [135]. In a molecular memory, a monolayer of molecules is sandwiched between a cross-point array of top and bottom electrodes as shown in Figure 20. The molecules are packed in a highly ordered way, with one end of the molecule electrically connected to the bottom electrode and the other end of the molecule connected to the top electrode, and this molecular component is described as a molecular switch [136]. Langmuir-Blodgett (LB) deposition is ideally suited for depositing the molecular layer for the fabrication of molecular memory devices [137,138]. Then, regarding the molecular memory operation, by applying a voltage between the electrodes, the conductivity of the molecules is altered, enabling data to be stored in a nonvolatile way. This process can then be reversed, and the data can be erased by applying a voltage to the opposite polarity of the memory cell. The increasing demand for nonvolatile electronic memories will grow rapidly in order to keep pace with the requirements for subsystems involved in flight demonstration projects and deep space operations. At the same time, mass, volume, and power must be minimized for mission affordability concerning these requirements; molecular memory could be a very promising candidate to fill this need.

Recently, Plafke has revealed clearly via an article that like most experimental technology that sounds so amazing that we want it right now, the molecular memory cell does not provide enough power for a commercial device [139]. This is currently only able to produce a 20% jump in conductivity. However, the area of molecular switching memory is promising, having eliminated the need for near-absolute zero temperatures and removed some of the constraints of the shape and number of layers of the molecule sheets which intend to convey that two of the biggest barriers are taken away. Thus, molecular memory requires strong attention to work over such issues and needs immediate amendment to see the possibility of a universal memory in the future.

#### ***MNW***

**Figure 21:**
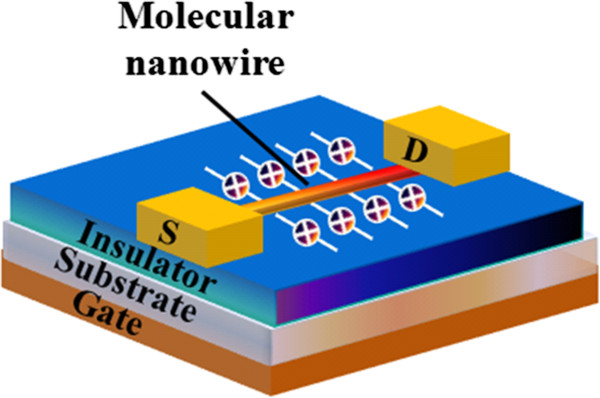
A MNW memory cell structure.

In the last two decades, an increasing interest is observed for electronics-related devices and the search for a universal memory data storage device that combines rapid *read* and *write* speeds, high storage density, and nonvolatility is driving the investigation of new materials in the nanostructured form [140]. As an alternative to the current Flash memory technology, a novel transistor architecture using molecular-scale nanowire memory cells holds the promise of unprecedently compact data storage. The molecular nanowire array (MNW) memory is fundamentally different from other semiconductor memories; information storage is achieved through the channel of a nanowire transistor that is functionalized with redox-active molecules rather than through manipulation of small amounts of charge. It is relatively slow and lacks the random access capability, wherein data that can be randomly read and written at every byte are being actively pursued. Figure 21 shows the schematic design of a MNW memory cell. Lieber, and Agarwal and Lieber have revealed that the nanowire-based memory technology is a powerful approach to assemble electronic/photonic devices at ultra-small scales owing to their sub-lithographic size, defect-free single-crystalline structure, and unique geometry [141,142]. Nanowires synthesized by chemical or physical processes are nearly perfect single-crystal structures with a small geometry and perfect surface. The channel of a nanowire transistor is functionalized with redox-active molecules. During programming, control of the voltage acting on the substrate is possible to change the oxidation and reduction states of the active molecules. Finally, by measurement of the conductance of the nanowire with the gate bias fixed at 0 V or a small voltage and from the hysteresis, the two states can be defined as a high-conductance ON state and a low-conductance OFF state. The MNW memory has advantages of low power dissipation, ultra-high density, simple fabrication process, 3-D structure, and multilevel storage, and it functions at the nanoscale with a few electrons but limited by low retention time parameter [143,144]. Moreover, the deposition of metals onto a monolayer of molecular wires can lead to low device yield, and this problem remains a major challenge [145]. However, mentioning the term emerging class memory, it could be expected that the MNW memory represents an important step towards the creation of molecular computers that are much smaller and could be more powerful than today's silicon-based computers.

#### ***SNW***

**Figure 22:**
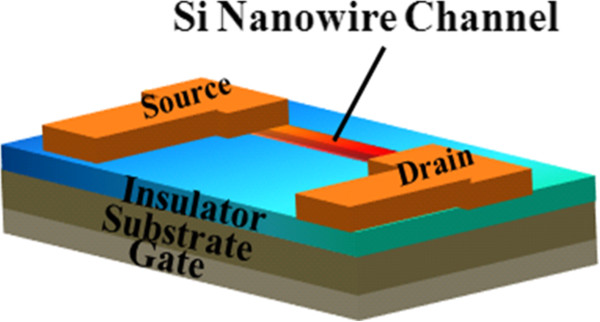
A bottom-gate FET-based nonvolatile SNW memory device.

Semiconductor memory is essential for information processing as a key part of silicon technology; semiconductor memory has been continuously scaled to achieve a higher density and better performance in accordance with Moore's law [146]. Flash memory may reach fundamental scaling limits, however, because a thick tunneling oxide is required to prevent charge leakage and achieve 10 years' retention. As Flash memory approaches its scaling limit, several alternative strategies have been proposed to extend or replace the current Flash memory technology [147]. These approaches are revolutionary, but major challenges must be overcome to achieve small memory size and aggressive technology design architecture. In addition to the engineering of trapping layers, the device performance can also be improved by using innovative nonplanar channel geometries. Among the various nanostructure materials, semiconductor nanowire memory (SNW) has induced great scientific interest as possible building blocks for future nanoelectronic circuitry. In a SNW memory device, nanowires are integrated with SONOS technology. The basic schematic design of SNW is depicted in Figure 22. The SNW memory shows high mobility, less power dissipation, and high performance. Moreover, being 3-D-stacked, the SNW memory enhances cell density and data capacity without relying on advances in process technology. The nanowire-based memory device can store data electrically and is nonvolatile, meaning it retains data when the power is turned off, like the silicon-based Flash memory found in smart phones and memory cards [148], with minimal increase in chip size. In addition, the SNW device exhibits reliable write/read/erase operations with a large memory window and high on-to-off current ratio, which are highly advantageous for applications in nonvolatile memory [149]. The SNW memory cannot hold data as long as the existing Flash, but it is slower and has fewer rewrite cycles and it could potentially be made smaller and packed together more densely. And its main advantage is that it can be made using simple processes at room temperature, which means that it can be deposited even on top of flexible plastic substrates [150]. The SNW could, for instance, be built into a flexible display and could be packed into smaller spaces inside cell phones, MP3 players, plastic RFID tags, and credit cards.

#### ***NRAM***

**Figure 23:**
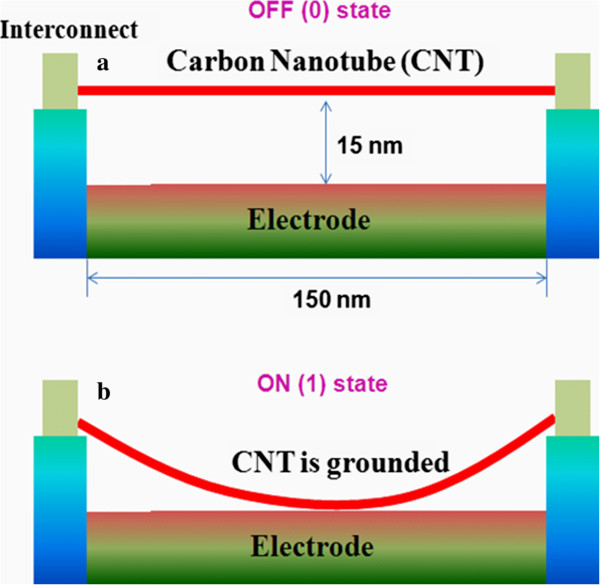
NRAM structure with (a) OFF state and (b) ON state.

NRAM is a carbon nanotube (CNT)-based memory, which works on a nanomechanical principle, rather than a change in material properties [151]. NRAM uses carbon nanotubes for the bit cells, and the 0 or 1 is determined by the tube's physical state: up with high resistance, or down and grounded. NRAM is expected to be faster and denser than DRAM and also very scalable, able to handle 5-nm bit cells whenever CMOS fabrication advances to that level. It is also very stable in its 0 or 1 state. Produced by Nantero, these memories consist of the structure shown in Figure 23a with an array of bottom electrodes covered by a thin insulating spacer layer [152]. CNTs are then deposited on the spacer layer, leaving them freestanding above the bottom electrodes. Unwanted CNTs are removed from the areas around the electrode, with top contacts and interconnects deposited on top of the patterned CNT layer. During the time that the CNTs are freestanding, there is no conduction path between the bottom and top electrodes and hence the memory cell is in the *OFF* state. However, if a large enough voltage is applied over the cell, the nanotubes are attracted to the bottom electrode where they are held in place by van der Waals forces [153]. Due to the conductive nature of the CNTs, the electrodes are now connected and the cell reads the low conductivity *ON* state as shown in Figure 23b. The *OFF* state can be returned by repelling the nanotubes with the opposite electrode polarity. Nonvolatility is achieved due to the strength of the van der Waals forces overcoming the mechanical strain of the bent nanotubes, hence holding the cell in the *ON* state. NRAM offers the possibility of a simple cell architecture, which could operate at much higher speeds than the conventional Flash and with low power use. Cui et al. reported CNT memory devices exhibiting an extraordinarily high charge storage stability of more than 12 days at room temperature [154]. However, as NRAM is based on CNTs, it suffers from fabrication problems that are inherent in carbon nanotube-based devices. The issues include the cost and fabrication complexity of producing the CNTs, ensuring uniform dispersions of nanotubes, and difficulties in removing nanotubes from the unwanted positions on the substrate.

#### ***Millipede memory***

**Figure 24:**
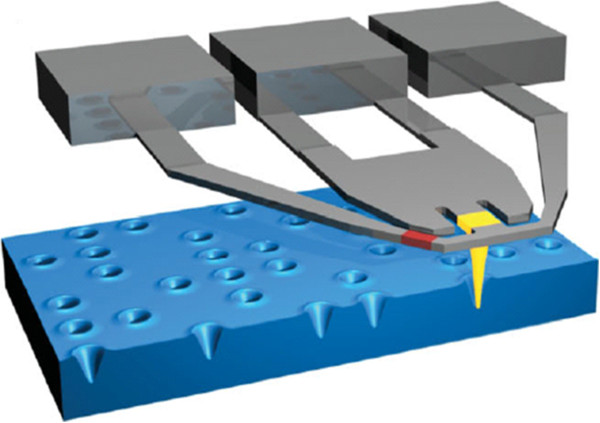
**Schematic layout of the millipede cantilever/tip in contact with the data storage medium.** Adopted from ref. [157].

In 2002, IBM developed a punch card system known as Millipede, which is a nonvolatile computer memory stored in a thin polymer sheet with nanoscopic holes to provide a simple way to store binary data [155]. It can store hundreds of gigabytes of data per square centimeter. However, the polymer reverts to its pre-punched form over time, losing data in the process. Millipede storage technology is being pursued as a potential replacement for magnetic recording in hard drives, at the same time reducing the form factor to that of Flash media. The prototype's capacity would enable the storage of 25 DVDs or 25 million pages of text on a postage stamp-sized surface and could enable 10 GB of storage capacity on a cell phone. Millipede uses thousands of tiny sharp points (hence the name) to punch holes in a thin plastic film. Each of the 10-nm holes represents a single bit. The pattern of indentations is a digitized version of the data. The layout of the millipede cantilever/tip in contact with the data storage medium is shown in Figure 24. According to IBM, Millipede can be thought of as a nanotechnology version of the punch card data processing technology developed in the late nineteenth century [156]. However, there are significant differences: Millipede is rewritable, and it may eventually enable storage of over 1.5 GB of data in a space no larger than a single hole in the punch card. Storage devices based on IBM's technology can be made with existing manufacturing techniques, so they will not be expensive to make. According to P. Vettiger, head of the Millipede project, there is not a single step in fabrication that needs to be invented. Vettiger predicts that a nanostorage device based on IBM's technology could be available as early as 2005 [155]. Now, researchers at IBM's Zurich Research Laboratory in Switzerland have clocked the rate of data loss. They have calculated that at 85°C, a temperature often used to assess data retention, it would lose just 10% to 20% of information over a decade, comparable to Flash memory [157].

#### ***WORM memory based on DNA biopolymer nanocomposite***

**Figure 25:**
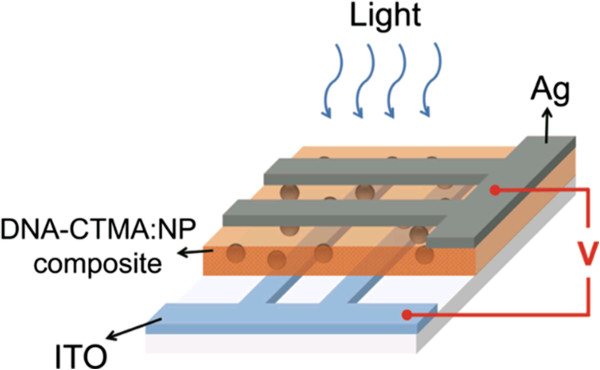
**Schematic design of a memory device consisting of a thin DNA biopolymer film sandwiched between electrodes.** The memory switching effect is activated upon light irradiation. Adopted from ref. [161].

The use of DNA is well known as a good model for metal NP synthesis due to its affinity to the metal ions [158]. In recent years, DNA has also been shown to be a promising optical material with the material processing fully compatible with conventional polymer for thin-film optoelectronic applications [159,160]. Researchers from National Tsing Hua University in Taiwan and the Karlsruhe Institute of Technology in Germany have created a DNA-based memory device, that is, write-once-read-many-times (WORM), that uses ultraviolet (UV) light to encode information [161]. The device consists of a single biopolymer layer sandwiched between electrodes, in which electrical bistability is activated by *in situ* formation of silver nanoparticles embedded in a biopolymer upon light irradiation (Figure 25). The device functionally works when shining UV light on the system, which enables a light-triggered synthesis process that causes the silver atoms to cluster into nanosized particles and readies the system for data encoding. For some particular instance, the team has found that using DNA may be less expensive to process into storage devices than using traditional, inorganic materials like silicon, the researchers say [161,162]. They said that when no voltage or low voltage is applied through the electrodes to the UV-irradiated DNA, only a low current is able to pass through the composite; this corresponds to the ‘OFF’ state of the device. But the UV irradiation makes the composite unable to hold a charge under a high electric field, so when the applied voltage exceeds a certain threshold, an increased amount of charge is able to pass through. This higher state of conductivity corresponds to the ‘ON’ state of the device. The team found that this change from low conductivity (‘OFF’) to high conductivity (‘ON’) was irreversible: once the system had been turned on, it stayed on, no matter what voltage the team applied to the system. Once information is written, the device appears to retain that information indefinitely. The researchers hope that the technique will be useful in the design of optical storage devices and suggest that it may have plasmonic applications as well. Consequently, WORM memories based on DNA a biopolymer nanocomposite have emerged as an excellent candidate for next-generation information storage media because of their potential application in flexible memory devices. This work combines new advances in DNA nanotechnology with a conventional polymer fabrication platform to realize a new emerging class of DNA-based memory.

#### ***QD memory***

**Figure 26:**
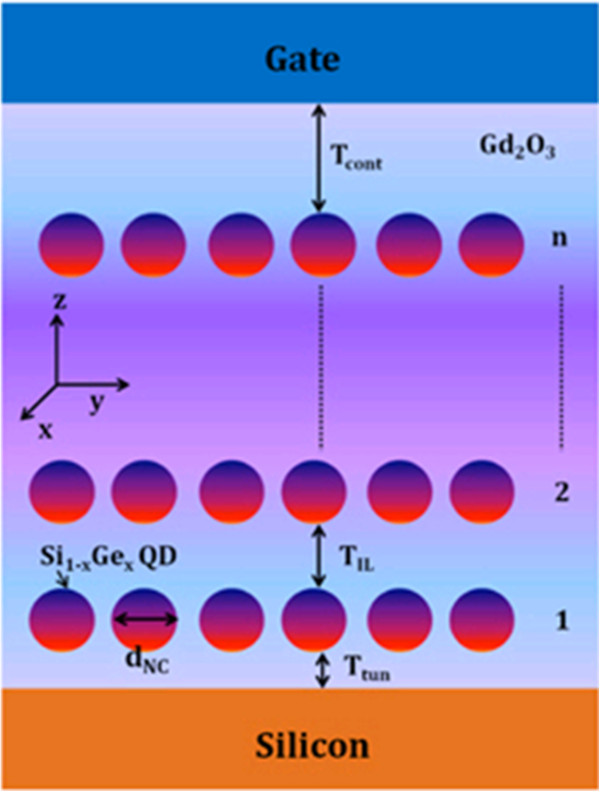
**Structure of quantum dot memory.** Adopted from ref. [168].

Memory made from tiny islands of semiconductors - known as quantum dots - could fill a gap left by today's computer memory, allowing storage that is fast as well as long lasting. Researchers have shown that they can write information into quantum dot memory in just nanoseconds. Memory is divided into two forms: DRRAM and Flash [163,164]. Computers use DRAM, for short-term memory, but data does not persist for long and must be refreshed over 100 times per second to maintain its memory. On the other hand, Flash memory, like that used in memory cards, can store data for years without refreshing but writes information about 1,000 times slower than DRAM. New research shows that memory based on quantum dots can provide the best of both: long-term storage with write speeds nearly as fast as DRAM. A tightly packed array of tiny islands, each around 15 nm across, could store 1 terabyte (1,000 GB) of data per square inch, the researchers say. Dieter Bimberg and colleagues at the Technical University of Berlin, Germany, with collaborators at Istanbul University, Turkey, demonstrated that it is possible to write information to the quantum dots in just 6 ns [165,166]. The key advantages of quantum dot (QD) NVMs are the high read/write speed, small size, low operating voltage, and, most importantly, multibit storage per device. However, these features have not been realized due to variations in dot size and lack of uniform insulator cladding layers on the dots [167]. Incorporating QDs into the floating gate results in a reduction in charge leakage and power dissipation with enhanced programming speed. Researchers in India and Germany have now unveiled the memory characteristics of silicon and silicon-germanium QDs embedded in epitaxial rare-earth oxide gadolinium oxide (Gd_2_O_3_) grown on Si (111) substrates as shown in the DQM structure in Figure 26. Multilayer Si as well as single-layer Si_1 − *x*_Ge_*x*_ (where *x* = 0.6) QDs show excellent memory characteristics, making them attractive for next-generation Flash-floating-gate memory devices [168,169].

#### ***3-D cross-point memory***

**Figure 27:**
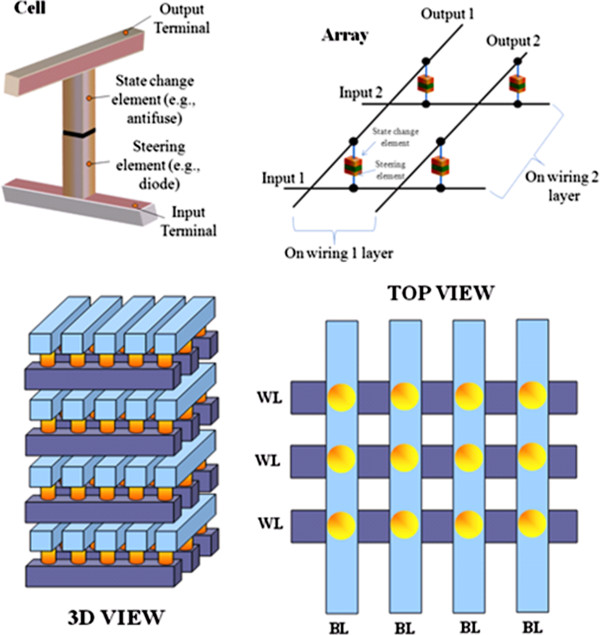
**The basic design of a 3-D cell that consists of a vertical diode in series.** (top) Side view, (bottom right) top view, and (bottom left) 3-D view.

Memory producers are also trying to develop alternative technologies that may be scalable beyond 20-nm lithography. For true scalability beyond 20-nm technology nodes, it is necessary to design a cross-point memory array which does not require diodes for access elements [170]. The cross-point memory architecture could be designed such that it can be easily fabricated in multiple layers to form a stacked 3-D memory [171]. The 3-D technology has brought to high volume an NVM where arrays of memory cells are stacked above control logic circuitry in the third dimension, and stacking 3-D memory directly over CMOS allows for high array efficiency and very small die size [172]. The 3-D technology uses no new materials, processes, or fabrication equipment, which control logic circuitry composed of typical CMOS. The memory construction uses typical back-end processing tools, and each memory layer is a repeat of the layers below it. The basic design of the 3-D cell consists of a vertical diode in series with a memory element as shown in Figure 27. Building integrated circuits vertically allows for a reduced chip footprint when compared to a traditional 2-D design, by an approximate factor of the number of layers used. This offers significant advantages in terms of reduced interconnect delay when routing to blocks that otherwise would have been placed laterally. The process for the 2-D cross-point array can be built into a multilayer 3-D architecture. Traditionally, a 3-D integrated circuit (3-D-IC) has used more than one active device layer. While resistance-change memory cells are not active devices, they function as rectifying devices in design. Further characterization of the resistance-change material is also necessary in order to guarantee that the 3-D cross-point memory will be practical for data storage. Also, the scalability of metal-oxide resistance-change materials beyond 20-nm technology nodes still needs to be studied. Moreover, the programming operation is expected to be competitive with both NAND and NOR Flash in terms of speed because of the relatively low voltage requirements of resistance-change materials. If the peripheral circuitry for accommodating the write operation can be made sufficiently compact, then the 3-D cross-point memory will indeed be a viable replacement for NAND and NOR Flash in future process generations.

#### ***TFM***

**Figure 28:**
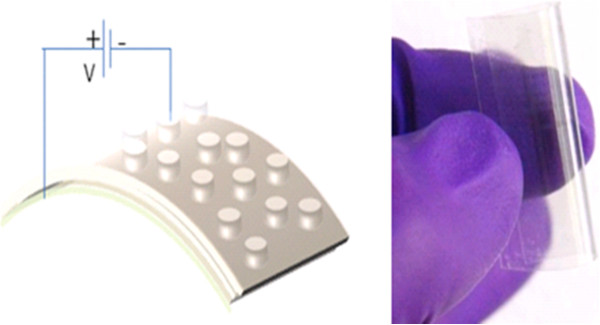
**A schematic design of FT-RRAM and a flexible, transparent memory chip image created by researchers at Rice University.** Reproduced from Tour Lab/Rice University.

Transparent and flexible electronics (TFE) is, today, one of the most advanced topics for a wide range of device applications, where the key component is transparent conducting oxides (TCOs), which are unique materials that oxides of different origin play an important role, not only as a passive component but also as an active component [173]. TFE is an emerging technology that employs materials (including oxides, nitrides, and carbides) and a device for the realization of invisible circuits for implementing next-generation transparent conducting oxides in an invisible memory generation [174]. In general, the TF-RRAM device is based on a capacitor-like structure (e.g., ITO/transparent resistive material/ITO/transparent and flexible substrate), which provides transmittance in the visible region [175]. For such new class of memory technology, data retention is expected to be about 10 years. The basic structural design of the new memory chips is configured, namely with two terminals per bit of information on a transparent and flexible substrate rather than the standard three terminals per bit on a rigid and opaque substrate (Figure 28). They are much better suited for the next revolution in electronic 3-D memory than Flash memory. These new memory chips that are transparent, are flexible enough to be folded like a sheet of paper, shrug off 1,000°F temperatures twice as hot as the max in a kitchen oven, and survive other hostile conditions could usher in the development of next-generation Flash-competitive memory for tomorrow's keychain drives, cell phones, and computers, a scientist reported today. Speaking at the 243rd National Meeting and Exposition of the American Chemical Society, the world's largest scientific society, he said that devices with these chips could retain data despite an accidental trip through the drier or even a voyage to Mars. And with a unique 3-D internal architecture, the new chips could pack extra gigabytes of data while taking up less space [176]. Despite the recent progress in TF-RRAM, it needs lots of work to satisfy the dual requirements of resistance to repeated bending stress and transparent properties. Thus, it is supposed that an achievement of such TF-RRAM device will be the next step towards the realization of transparent and flexible electronic systems. We hope that FT-RRAM devices will mark a milestone in the current progress of such unique and invisible electronic systems in the near future.

#### ***1T1R-RRAM***

**Figure 29:**
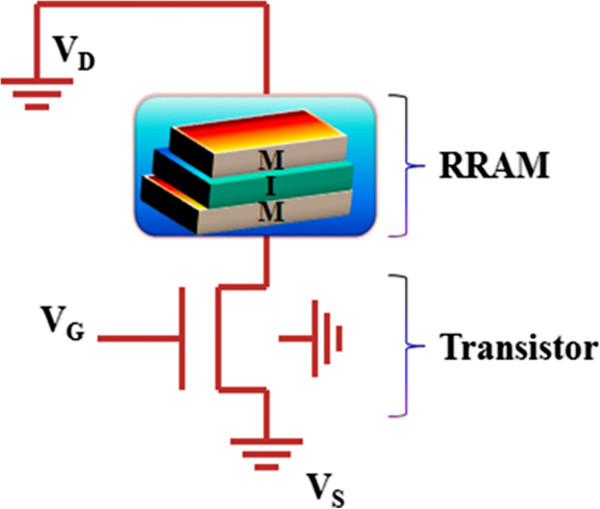
The basic cell structure of 1T1R-RRAM.

One-transistor one-resistor (1T1R)-RRAM is also one class of emerging memory technology with impressive characteristics. It meets the demands for next-generation memory systems. It is expected that 1T1R-RRAM could be able to meet the demand of high-speed (e.g., performance) memory technology. The 1T1R structure is chosen because the transistor isolates current to cells, which are selected from cells which do not. The basic cell structure of 1T1R is depicted in Figure 29. 1T1R-RRAM consists of an access transistor and a resistor as a storage element. Zangeneh and Joshi have also mentioned that the 1T1R cell structure is similar to that of a DRAM cell in that the data is stored as the resistance of the resistor and the transistor serves as an access switch for reading and writing data [177,178]. In reference to this, they revealed the 1T1R cell as the basic building block of a NVRRAM array as it avoids sneak path problem to ensure reliable operation. Moreover, the 1T1R structure is more compact and may enable vertically stacking memory layers, ideally suited for mass storage devices. But, in the absence of any transistor, the isolation must be provided by a ‘selector’ device, such as a diode, in series with the memory element, or by the memory element itself. Such kinds of isolation capabilities have been inferior to the use of transistors, limiting the ability to operate very large RRAM arrays in 1T1R architecture. 1T1R memory polarity can be either binary or unary. Bipolar effects cause polarity to reverse reset operation to set operation. Unipolar switching leaves polarity unaffected but uses different voltages.

#### ***MTM, PFRAM, SPBMM, and CMORRAM - future alternate NVMs***

Other potential emerging classes of memory technologies, we are describing in short, are molecular tunnel memory (MTM), polymeric ferroelectric RAM (PFRAM), spin-polarized beam magnetic memory (SPBMM), light memory, and complex metal-oxide RRAM (CMORRAM). We can say that these are sister memory technologies of molecular memory, ferroelectric/polymer memory, magnetic memory, and metal-oxide RRAM, respectively. Although these new technologies will almost certainly result in more complex memory hierarchies than their family memories, they are likely to allow the construction of memory chips that are nonvolatile, have low energy, and have density and development close to or better than those of DRAM chips, with improved performance and allowing memory systems to continue to scale up.

## Conclusions

This article reviewed the historical development to the recent advancement on memory architecture and scaling trend of several conventional types of Flash within the MOS family and projected their future trends. With great progress being made in the emerging memory technologies, current trends and limitations were discussed before leading to some insight into the next generation of memory products. For the past three and a half decades in existence, the family of semiconductor memories has expanded greatly and achieved higher densities, higher speeds, lower power, more functionality, and lower costs. In the past 40 to 50 years, NVSM has grown from the FG concept to FAMOS, SAMOS, Flash memory, multilevel cells, RRAM, 3-D structures, and TF-RRAM. Since 1990, NVSM is an inspired technology, which has ushered in the digital age, enabled the development of all modern electronic systems, and brought unprecedented benefit to humankind. At the same time, some of the limitations within each type of memory are also becoming more realized. As the device dimension is reduced to the deca-nanometer regime, NVSM faces many serious scaling challenges such as the interface of neighboring cells, reduction of stored charges, and random telegraph noise. As such, we hope and are confident that there are several emerging technologies aiming to go beyond those limitations and potentially replace all or most of the existing semiconductor memory technologies to become a USM. Despite these limitations, the field of conventional semiconductor memories would continue to flourish and memory device scientists will find the way to meet these challenges and may even develop a ‘unified memory’ with low cost, high performance, and high reliability for future electronic systems. Progress towards a viable new resistive memory technology relies on fully understanding the mechanisms responsible for switching and charge transport, the failure mechanisms, and the factors associated with materials reliability. Moreover, the development of current redox-based resistive switching will help to improve our old technologies, and further research will produce more impressive results that will benefit industries and society to improve the quality of life for billions of people around the world.

## Competing interests

The authors declare that they have no competing interests.

## Authors' contributions

JSM designed the structure and modified the manuscript. SMS and TYT participated in the sequence alignment and editing the manuscript. UC participated in its design and coordination and helped to draft the manuscript. All authors read and approved the final manuscript.

## Authors' information

JSM received his bachelor's degree (Physics Honors) from the Department of Physics, Aligarh Muslim University, Aligarh, India, and Master's degree in solid-state technology from the Department of Physics and Meteorology, Indian Institute of Technology (IIT), Kharagpur, India, in 2007. He received his Ph.D. degree in Nanotechnology from National Chiao Tung University (NCTU), Taiwan, in February 2012. From March 2012 to December 2013, he has been a Postdoctoral Research Associate in the Department of Photonics and Display Institute, NCTU, Taiwan. He is currently a Postdoctoral Research Associate in the Department of Electronics and the Institute of Electronics, NCTU, Taiwan. His current research interests include designing, fabrication, and testing of a transparent and flexible random-access memory (RRAM) for application in invisible and rollable nonvolatile memory devices. He has published various research papers in reputed journals and presented his research in international conferences over flexible substrate-based thin-film transistors and capacitor devices for their applications in display and RF identification tags.

SMS received his B.S. degree from National Taiwan University, M.S. degree from the University of Washington, and Ph.D. degree from Stanford University, all in Electrical Engineering. He was with Bell Telephone Laboratories from 1963 to 1989 as a member of the Technical Staff. He joined National Chiao Tung University (NCTU) from 1990 to 2006 as a Distinguished Professor. At present, he is a National Endowed Chair Professor at NCTU. He has served as a Visiting Professor or Consulting Professor to many academic institutions including the University of Cambridge, Delft University, Beijing Jiaotong University, Tokyo Institute of Technology, Swiss Federal Institute of Technology, and Stanford University. He has made fundamental and pioneering contributions to semiconductor devices, especially metal-semiconductor contacts, microwave devices, and submicron MOSFET technology. Of particular importance is his coinvention of the *nonvolatile semiconductor memory* (NVSM) which has subsequently given rise to a large family of memory devices including Flash memory and EEPROM. The NVSM has enabled the development of *all modern electronic systems* such as the digital cellular phone, ultrabook computer, personal digital assistant, digital camera, digital television, smart IC card, electronic book, portable DVD, MP3 music player, antilock braking system (ABS), and Global Positioning System (GPS). He has authored or coauthored over 200 technical papers. He has written and edited 16 books. His book *Physics of Semiconductor Devices* (Wiley, 1969; 2nd ed, 1981; 3rd ed, 2007) is one of the *most* cited works in contemporary engineering and applied science publications (over 24,000 citations according to ISI Press). He has received the IEEE J.J. Ebers Award, the Sun Yat-sen Award, the National Endowed Chair Professor Award, and the National Science and Technology Prize. He is a Life Fellow of IEEE, an Academician of the Academia Sinica, a foreign member of the Chinese Academy of Engineering, and a member of the US National Academy of Engineering.

UC received his MS degree in Solid State Electronics from the Indian Institute of Technology, Roorkee, India, in 2010. He is currently a Ph.D. candidate of the Institute of Electronics, National Chiao Tung University, Taiwan. He is currently working on the fabrication and characterization of resistive switching memory devices with a focus on memory structure innovations and reliability.

TYT is now a Lifetime Chair Professor in the Department of Electronics Engineering, National Chiao Tung University. He was the Dean of the College of Engineering and Vice Chancellor of the National Taipei University of Technology. He received numerous awards, such as the Distinguished Research Award from the National Science Council, Academic Award of the Ministry of Education, National Endowed Chair Professor, and IEEE CPMT Outstanding Sustained Technical Contribution Award.
